# Protein conformational transition microenvironment in silk fibroin hydrogels: proliferation and chondrogenesis of encapsulated stem cells

**DOI:** 10.1093/rb/rbaf102

**Published:** 2025-10-01

**Authors:** Weikun Zhao, Guolong Cai, Jiayao Qian, Jingjing Geng, Xiang Yao, Yaopeng Zhang

**Affiliations:** State Key Laboratory of Advanced Fiber Materials, College of Materials Science and Engineering, Shanghai Engineering Research Center of Nano-Biomaterials and Regenerative Medicine, Donghua University, Shanghai 201620, P.R. China; State Key Laboratory of Advanced Fiber Materials, College of Materials Science and Engineering, Shanghai Engineering Research Center of Nano-Biomaterials and Regenerative Medicine, Donghua University, Shanghai 201620, P.R. China; State Key Laboratory of Advanced Fiber Materials, College of Materials Science and Engineering, Shanghai Engineering Research Center of Nano-Biomaterials and Regenerative Medicine, Donghua University, Shanghai 201620, P.R. China; State Key Laboratory of Advanced Fiber Materials, College of Materials Science and Engineering, Shanghai Engineering Research Center of Nano-Biomaterials and Regenerative Medicine, Donghua University, Shanghai 201620, P.R. China; State Key Laboratory of Advanced Fiber Materials, College of Materials Science and Engineering, Shanghai Engineering Research Center of Nano-Biomaterials and Regenerative Medicine, Donghua University, Shanghai 201620, P.R. China; State Key Laboratory of Advanced Fiber Materials, College of Materials Science and Engineering, Shanghai Engineering Research Center of Nano-Biomaterials and Regenerative Medicine, Donghua University, Shanghai 201620, P.R. China

**Keywords:** silk fibroin hydrogel, material stiffening and shrinkage, cell proliferation, cell chondrogenesis, conformational transition microenvironment

## Abstract

Chemically crosslinked silk fibroin (SF) hydrogels exhibit excellent extracellular matrix-mimicking features and tunable mechanical characteristics, making them highly promising for 3D cell culture and tissue engineering. However, the protein segments within SF hydrogels can spontaneously undergo a conformational transition from random coil to *β*-sheet, inducing dynamic changes in the material’s mechanical properties and pore structures. Such dynamical material cues could probably have significant effects on cell behaviors, thus inducing a kind of unknown influence which cannot be ignored when applying these hydrogels in 3D cell culture and tissue repair. Based on this, the current research seeks to clearly reveal the impacts of the protein conformational transition microenvironment within SF hydrogels on the proliferation and chondrogenic differentiation of encapsulated stem cells. To this end, this study successfully constructed a series of SF hydrogels with highly similar initial properties but different conformational transition rates, which was enabled by modulating the uniformity of the chemical crosslinking points while fixing the similar crosslinking density. Results showed that the SF hydrogel with lower uniformity of crosslinking points exhibited faster conformational transition rates, and *vice versa*. Encapsulated mesenchymal stem cells’ responses further clearly illustrated that the protein conformational transition microenvironment in SF hydrogels could obviously regulate cell proliferation and chondrogenesis. Specifically, a relatively slower conformational transition rate was more favorable for encapsulated cell proliferation, whereas a moderate transition rate was more beneficial for encapsulated cell chondrogenesis. Related research is expected to expand the knowledge and understanding of the impacts of dynamical protein conformational transition microenvironment on cell behavior within hydrogels, and provide valuable insights for the development of efficient SF-based cell culture matrices and cartilage scaffolds.

## Graphical abstract

**Figure:**
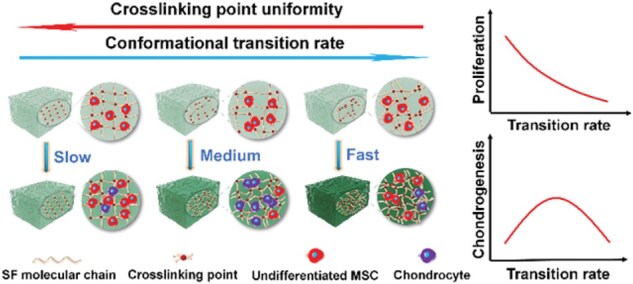

## Introduction

Silk fibroin (SF) is a natural protein that can be feasibly produced on a large scale. This raw material has garnered significant interest from biomaterial scientists and entrepreneurs because of its excellent comprehensive performance, including good biocompatibility, adjustable biodegradability, ease of processing and Food and Drug Administration (FDA) approval [1–6]. Among various types of SF-based biomaterials, the chemical crosslinked SF hydrogels exhibit promising extracellular matrix (ECM) mimicking property, suitable pore structures and a broad range of tunable mechanical properties [7–9]. In recent years, numerous studies have investigated the application of chemical crosslinked SF hydrogels in 3D cell culture [10–14], controlled release of drugs and bioactive factors [15], artificial corneas [16] and varied tissue engineering scaffolds [8, 17–21], etc, demonstrating their strong performance and substantial potential for biomedical applications. Specifically, most chemical crosslinked SF hydrogels are formed through the crosslinking reaction of active functional groups in the hydrophilic segments of SF molecules [3]. It has been preliminarily reported that the proteins in these SF hydrogels spontaneously undergo a conformational transition process from random coil to *β*-sheet conformation under *in vitro* cell culture or *in vivo* implant conditions [22, 23], probably owing to the self-assembly of hydrophobic segments in SF molecular chains. This conformational transition significantly alters the pore structures, mechanical properties and transparency of the hydrogels [14, 24]. Classical studies in the field of cell-material interactions have demonstrated that such material properties, particularly the pore size and modulus, significantly influenced cell behaviors such as adhesion, proliferation and differentiation [3, 25–29].

In addition, the dynamic cues, such as material degradation and dynamical mechanical stimulation, can also generate profound effects on cell function regulation [10, 17, 30–32]. Consequently, the protein conformational transitions in SF hydrogels may also have a significant impact on cell behaviors, leading to an important unknown effect on their applications in 3D cell culture and tissue repair. Therefore, it is valuable to investigate the related effects on cell behaviors by constructing SF hydrogels with varied transition rates of protein conformation.

Until now, a few researchers have attempted to develop SF-based hydrogels with varied transition rates of protein conformation. For example, Mahajan *et al.* [23] created several composite hydrogels with varied protein conformational transition rates by combining SF with carboxymethyl cellulose and gelatin. However, this approach relies on altering the ratios of SF and other components, leading to significant variations in the initial chemical composition and compressive modulus of the hydrogels. Furthermore, Cai *et al.* [14] developed a strategy based on changing the chemical crosslinking density of hydrogels, using SF as the sole component to construct hydrogels with varied transition rates of protein conformation. This reported strategy [14] effectively eliminates the interference of different initial chemical components, while the different crosslinking density leads to significant differences in the initial modulus and average pore size of hydrogels. In order to more clearly reveal the conformational transition microenvironment effects on cell behaviors within SF hydrogels, it is crucial to construct SF hydrogels with equivalent initial features but different protein conformational transition rates.

**Figure 1.**
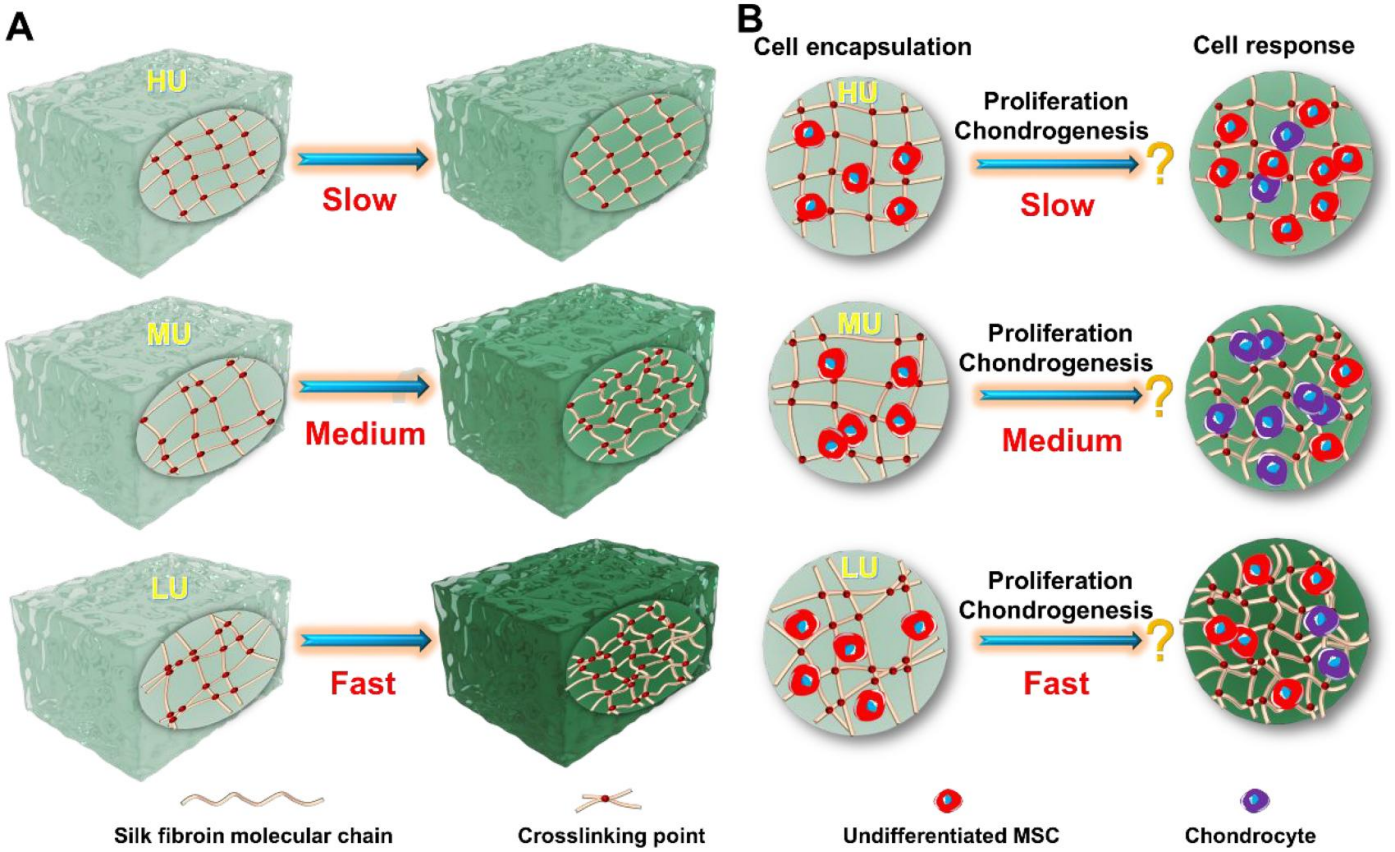
Schematic presentation of the control of protein conformational transition in SF hydrogels and the corresponding effects on encapsulated cell behaviors. (**A**) Regulation of the conformational transition rates in SF hydrogels by adjusting the uniformity degrees of crosslinking points; (**B**) Revealing of the effects of conformational transition microenvironments on the proliferation and chondrogenic differentiation of stem cells within hydrogels. HU, high uniformity of crosslinking points; MU, medium uniformity of crosslinking points; LU, Low uniformity of crosslinking points.

The corresponding SF hydrogel platforms reported thus far fail to meet the requirement for highly similar initial material properties. This failure could probably introduce non-negligible interference factors when investigating the impact of conformational transitions on cell behaviors. In the current literature, a novel strategy based on altering the uniformity degrees of crosslinking points while fixing the crosslinking density was proposed for constructing the desired SF hydrogel platforms with highly similar initial material features but different protein conformational transition rates, as schematically illustrated in Figure 1A. Theoretically, under similar crosslinking density, the hydrogel with low uniformity (LU) of crosslinking points has relative ‘longer’ free molecular chains compared to the hydrogel with high uniformity (HU) of crosslinking points. These ‘longer’ free chains are more feasible to undergo free movement and self-assembly, resulting in faster conformational transition rates compared to the HU group. In addition, the similar crosslinking density will guarantee the highly similar initial properties of SF hydrogels as much as possible. Moreover, mesenchymal stem cells (MSCs) were selected as a model cell to investigate how the protein conformational transition microenvironment in SF hydrogels influences the encapsulated cell proliferation and chondrogenesis, as schematically illustrated in Figure 1B.

More specifically, we choose two crosslinking induction systems with similar crosslinking mechanism to construct the related SF hydrogels. One is a visible light-inducing crosslinking system using tris(2,2-bipyridyl) dichlororuthenium(II) hexahydrate (Ru) and sodium persulfate (SPS) [33]. The other is a body temperature (37°C) inducing crosslinking system using horseradish peroxidase (HRP) and hydrogen peroxide (H_2_O_2_) [34]. Both of them rely on mild chemical reactions to form di-tyrosine, which establishes the crosslinking networks in SF hydrogels. The key difference between these two systems is that the molecular weight of HRP (Mw = 44 000 Da) is quite larger than that of Ru (Mw = 750 Da) [33]. And the reaction speed of Ru/SPS system is significantly faster than that of the HRP/H_2_O_2_ system [3]. Comprehensively, under the similar hydrogel fabrication condition, Ru could disperse more easily and uniformly into the hydrogel precursor system and trigger the reaction much more quickly compared to HRP, leading to a crosslinking network with a higher degree of uniformity. Furthermore, through the combination and dosage ratio regulation of Ru/SPS and HRP/H_2_O_2_, SF hydrogels with similar crosslinking density while different crosslinking uniformity degrees are expected to be constructed. Different from our previous literature [14] and other related report [23], this hydrogel construction strategy aims to minimize the impacts of initial material properties differences as much as possible, thus provides a robust material platform for accurately studying and confirming the effects of conformational transition microenvironments on encapsulated stem cell behaviors. The related research are anticipated to serve valuable reference for the controlling of conformational transition within SF hydrogels, and also offer important insights for the effective application of protein conformational transition in 3D cell culture, organoid construction and tissue repair.

## Materials and methods

### Materials

The cocoons of silkworm were obtained from Guizhou New Silk Road & Beautiful Life Technology Co., Ltd. HRP (295 U/mg) and Ru were obtained from Sigma, USA. Anhydrous sodium carbonate (Na_2_CO_3_), SPS, chloroform, isopropanol and 30% H_2_O_2_ (analytical grade) were sourced from Sinopharm Chemical Reagent Co., Ltd. Lithium bromide (LiBr, analytical grade) was provided by Shanghai Zhongli Industrial Co., Ltd. Dialysis bags were purchased from Shanghai Yuanju Biotechnology Co., Ltd. Phosphate-buffered saline (PBS), Dulbecco’s modified eagle medium (DMEM) with high glucose, DMEM with low glucose, penicillin-streptomycin solution (PS, 100 X), fetal bovine serum (FBS) and trypsin (0.25%) were obtained from Gibco, USA. RIPA lysis buffer and Cell Titer-Glo (CTG) reagent were purchased from Promega, USA. Calcein-AM and propidium iodide (CA/PI) working solutions were sourced from Shanghai Yeason Biotechnology Co., Ltd. Passage 0 (P0) rat bone marrow MSCs were obtained from Shanghai Yuchun Biotechnology Co., Ltd. Assay Buffer and TRIzol reagent were purchased from Shanghai Yuanye Biotechnology Co., Ltd. Ethanol, chloroform and isopropanol were obtained from Sinopharm Group Chemical Reagent Co., Ltd. The rabbit anti-Aggrecan antibody (primary antibody), rabbit anti-Collagen II antibody (primary antibody) and goat anti-rabbit IgG-HRP (secondary antibody) were purchased from Jiangsu Qinke Biological Research Center Co., Ltd. The rabbit anti-COMP antibody (primary antibody), mouse anti-GAPDH antibody (primary antibody) and goat anti-mouse IgG-HRP (secondary antibody) were purchased from Wuhan Sanying Biotechnology Co., Ltd. The rabbit anti-PRG4 antibody (primary antibody) was purchased from Wuhan Aibotai Biological Technology Co., Ltd.

### Preparation of SF aqueous solution and lyophilized powder

The preparation process of SF aqueous solution and powder could be referred from previous reports [14, 35, 36]. More specifically, silkworm cocoons were opened and the innermost and outermost layers were removed before cutting the remaining material into pieces. Then, 20 g of the cocoon pieces were placed in a 0.02 mol/L Na_2_CO_3_ solution and boiled for 40 min to remove the outer sericin. After that, the degummed silk was washed four times with deionized water and then air-dried. The dry degummed silk was further dissolved in a 40°C LiBr solution, with a ratio of 10 g degummed silk to 100 mL of LiBr solution (9 mol/L). After dissolution, the SF solution underwent filtration to eliminate impurities. Then, it was placed in a dialysis bag and dialyzed in deionized water at 4°C, with fresh water changed every 6 h. Once the conductivity of the SF solution in the dialysis bag reached below 4 μS/cm, the solution was concentrated to about 5 wt % by blowing air at 4°C, and then freeze-dried to obtain the corresponding lyophilized powder for subsequent usage.

### Preparation of SF hydrogels with highly similar initial properties while different conformational transition rates

#### Formulation exploration of HRP-SF and Ru-SF hydrogels with highly similar initial mechanical properties

In the preparation of HRP-SF hydrogels, the ratio of HRP to H_2_O_2_ was fixed at 1 U/mL: 2.45 × 10^−4^ mol/L according to literature [14]. For Ru-SF hydrogels, the ratio of Ru to SPS was set at 1 mol/L : 10 mol/L based on reported studies [10, 33]. During the formulation optimization process, the SF solution was fixed at 2 mL, and the amounts of HRP or Ru were adjusted to produce HRP-SF and Ru-SF hydrogels with different mechanical properties. The purpose of this experiment is to find the proper formulations of HRP-SF and Ru-SF hydrogels with highly similar initial mechanical properties.

The SF solution was prepared using DMEM medium as the solvent. Based on the precursor solution formulations in Table S1, Ru, SPS, HRP and H_2_O_2_ solutions were added to the SF solution. After thoroughly mixing, the crosslinking was carried out at 37°C under white light illumination (intensity: 30 mW cm^−2^) for 40 min to form the HRP-SF or Ru-SF hydrogels. The prepared Ru or HRP SF hydrogels were named based on the volume of Ru or HRP solution employed in Table S1. For example, the Ru35 reflects the SF hydrogel used 35 μL of Ru solution, and the HRP40 reflects the SF hydrogel used 40 μL of HRP solution.

The compressive modulus and strength of Ru-SF hydrogels with different Ru amounts (Ru35, Ru45, Ru55) and HRP-SF hydrogels with different HRP amounts (HRP40, HRP50, HRP60) were evaluated using an electronic universal testing equipment (Hengyu Instrument, HY-941). The results (see Figure S1A and B) showed that the compressive mechanical properties of Ru55 and HRP60, Ru45 and HRP50, and Ru35 and HRP40 were relatively similar. To minimize the potential negative effects on cells caused by excessive initiator concentrations, the two groups with higher initiator levels (Ru55 and HRP60, Ru45 and HRP50) were excluded from further experiments. Among the remaining groups (Ru35 and HRP40), although the compressive modulus and strength were similar, they still did not meet the precise matching requirement. Therefore, to achieve more accurate matching of the initial mechanical properties, the amount of HRP was further refined, and additional groups (HRP44 and HRP48) were also tested (Table S1). The compressive modulus and strength of these groups (Ru35, HRP40, HRP44 and HRP48) were shown in Figure S1A and B, which indicate that the Ru35 and HRP44 could exhibit a ‘precise match’ of initial compressive mechanical properties. The detailed stress–strain curves of Ru35 and HRP44 (Figure S1C) further confirmed this conclusion.

#### Preparation of SF hydrogels with highly similar initial properties while different conformational transition rates using the combination of HRP and Ru

The aforementioned explorations collectively demonstrate that the individual use of HRP44 and Ru35 can effectively produce SF hydrogels with highly similar initial mechanical properties, which indicate that the hydrogels formed by ‘11 quality units’ of HRP and ‘8.75 quality units’ of Ru can present highly similar initial properties. Based on the relevant research, we further designed and constructed SF hydrogels with highly similar initial properties while different conformational transition rates by adjusting the combination ratio of Ru and HRP according to their equivalent quality units. The formulations of the precursor solutions for these SF hydrogels are detailly listed in Table S2. The preparation process is as follows: Ru, SPS, HRP and H_2_O_2_ solutions were added to the SF solution according to the formulations in Table S2. After thoroughly mixing, the crosslinking was performed at 37°C under white light illumination (intensity 30 mW cm^−2^) for 40 min. With the increase of HRP in the precursor formulation, the resulting SF hydrogels were sequentially named as H-I, H-II, H-III, H-IV and H-V.

### Evaluation of the initial features of SF hydrogels

#### Characterization of the crosslinking densities of SF hydrogels

The SF hydrogel (H-I ∼ H-V) samples were gelled in a 24-well plate, with each well containing 2 mL of SF hydrogel precursor solution. According to the typical literatures [37, 38], the di-tyrosine bonds (crosslinking points) fluorescence intensity in the fabricated hydrogels was measured using a fluorescence spectrophotometer (Proteostasis Therapeutics, Inc., model QM/TM). During the detection, the excitation wavelength was set as 315 nm, and the emission fluorescence intensity at 410 nm could be used to reflect the relative amount of di-tyrosine bonds. Because the SF hydrogels induced by HRP and Ru systems both depend on the formation of di-tyrosine bonds, the measured fluorescence intensity at 410 nm of these hydrogels could be positively correlated with the crosslinking density of the corresponding hydrogels (H-I ∼ H-V).

#### Characterization of the mechanical properties of SF hydrogels

The SF hydrogels (H-I ∼ H-V) were fabricated identically to those in section Characterization of the mechanical properties of SF hydrogels. The stress–strain behaviors of the compressive properties of SF hydrogels (H-I ∼ H-V) were evaluated using an electronic universal testing equipment at a compression speed of 8 mm/min. The compressive modulus of the material was defined as the slope of the linear region of the stress–strain curve between 5% and 15% strain [8, 14]. The compressive strength was defined as the stress corresponding to 30% strain [14, 23].

#### Evaluation of the internal microstructure of SF hydrogels

The SF hydrogels (H-I ∼ H-V) were fabricated identically to those in section Characterization of the mechanical properties of SF hydrogels. The fabricated SF hydrogels (H-I ∼ H-V) were subjected to freeze-drying treatment, followed by brittle fracture of the dried samples using liquid nitrogen. After removing residues from the fracture surface with a gas flow, the samples were affixed to the surface of a conductive adhesive with the fracture surface facing upward. Subsequently, a gold layer was deposited onto the samples by sputter coating for 90 seconds at a current of 10 mA. Finally, at 1 kV accelerating voltage condition of a scanning electron microscope (SEM, Hitachi, FlexSEM 1000II), the corresponding internal microstructures were photographed.

### Characterization of the protein conformational transition processes and their influence on the material properties of SF hydrogel

#### Evaluation of the changes in β-sheet structure contents of SF hydrogels after different incubation times

The SF hydrogel samples were gelled in a 24-well plate, with a volume of 700 μL of hydrogel precursor solution in each well. The fabricated SF hydrogels were then incubated in DMEM medium and placed in a cell culture incubator (simulated cell culture conditions). After 1, 4, 7, 10 and 14 days of incubation, the SF hydrogels were subjected to freeze-drying and compression. Subsequent analysis was performed using the attenuated total reflectance mode of a Fourier-transform infrared (FT-IR) spectrometer (Thermo Fisher, Nicolette 6700). Further analysis involved the deconvolution of the absorption peaks in the infrared spectra between 1600 and 1700 cm^−1^ using the Peakfit software. The *β*-sheet structure content in the indicated sample was quantified by calculating the ratio of related peak areas, which equals to area between 1616 and 1637 cm^−1^ divided by the area between 1600 and 1700 cm^−1^ accordingly [34].

#### Characterization of the changes in light transmittance of SF hydrogels after different incubation times

The SF hydrogel (H-I ∼ H-V) samples were gelled in a 96-well plate, with a volume of 100 μL of hydrogel precursor solution in each well. The samples were then incubated in DMEM medium and placed in a cell culture incubator. The initial absorbance of each SF hydrogel (after 1 day of incubation) was recorded at 620 nm by a microplate reader (Thermo Fisher, Multiskan FC) and was defined as OD_0_. Thereafter, the absorbance of indicated hydrogels was monitored daily from 1 ∼ 14 days of incubation and was defined as OD_T_. In this experiment, the change rate in the optical density (OD) of the SF hydrogels could serve as a quantitative light transmittance change indicator of the hydrogel. The formula for calculating the change rate of OD is as follows:


$${\mathrm{OD}}_{\mathrm{change}\;\mathrm{rate}}=\frac{{\mathrm{OD}}_{T}-{\mathrm{OD}}_{0}}{{\mathrm{OD}}_{0}}\times 100\%$$

#### Characterization of the changes in macroscopic state of SF hydrogels after different incubation times

Typical SF hydrogels (H-I, H-III and H-V) were fabricated in a 24-well plate with approximately 600 μL of precursor solutions in each well. The hydrogels were subsequently incubated in DMEM medium and placed in a cell culture incubator. After 1, 7 and 14 days of incubation, the samples were removed and placed onto a substrate marked with ‘SFH’ for photography to qualitatively reflect the transparency and light transmittance changes of the typical hydrogels.

#### Evaluation of the changes in microstructure of SF hydrogels after varied incubation times

SF hydrogels (H-I ∼ H-V) were fabricated and then incubated in DMEM medium and placed in a cell culture incubator. After 1, 4, 7, 10 and 14 days of incubation, the SF hydrogels were subjected to freeze-drying, followed by brittle fracture using liquid nitrogen. After removing residues from the fracture surface with a gas flow, the samples were affixed to the surface of a conductive adhesive with the fracture surface facing upward. Subsequently, at a current of 10 mA, the samples were sputter-coated with gold for 1.5 min. The internal microstructure properties of the samples were imaged by SEM (Hitachi, FlexSEM 1000II).

#### Characterization of the changes in compressive mechanical properties of SF hydrogels after varied incubation times

The SF hydrogels (H-I ∼ H-V) were all fabricated into cylinders measuring 15 mm in diameter and 8 mm in height. The hydrogels were then incubated in DMEM medium and placed in a cell culture incubator. After 1, 4, 7, 10 and 14 days of incubation, the corresponding stress–strain behaviors of each hydrogel were detected by an electronic universal testing equipment. Finally, the specific compressive modulus and strength of the hydrogels were determined from the detected stress–strain curves using the methods described in section Characterization of the mechanical properties of SF hydrogels.

### Stem cell culture and passage

Rat bone marrow MSCs (passage 0) cultured to about 80% confluence in a T25 culture flask were purchased. Then, cell passaging was conducted. Specifically, the culture medium was aspirated from the T25 culture flask, and 1 ml of pre-warmed (37°C) trypsin was added. The flask was then transferred into a cell culture incubator (37°C, 5% CO_2_). Immediately following 1.5 min incubation, 2 ml of cell culture medium (V_DMEM_: V_FBS_: V_PS_=100:10:1) was added to terminate the enzymatic digestion. The MSCs obtained after centrifugation were subsequently transferred to a T75 culture flask for further cultivation (37°C, 5% CO_2_), with fresh culture medium (15 mL) being changed every two days. When the MSCs reached 80% confluence in the T75 flask, passaging (from one T75 flask to three T75 flasks) was again performed to achieve desired number of stem cells. The MSCs used in the following experiments were the cells from passage 4 to passage 6 (P4–P6).

### Cultivation of stem cells in the SF hydrogels (H-I ∼ H-V)

SF lyophilized powder was dissolved in DMEM medium to prepare a 6% SF solution. Then, sterilize the corresponding SF solution using filters with 0.22 μm pores. The cell suspension was mixed with an equal volume of DMEM medium. The sterilized Ru solution, SPS solution, HRP solution and H_2_O_2_ solution were also prepared by DMEM medium and added to the mixture according to the formulation in Table S2. The resulting mixture was thoroughly mixed with proper cell suspension to obtain the MSCs-containing SF hydrogel precursor solutions. The corresponding precursor solutions were added to a 12-well plate (600 μL/well, with an MSCs density of 8 × 10^6^ cells/mL) and exposed to white light for 40 min at 37°C to form the MSCs encapsulated hydrogels of H-I ∼ H-V. The encapsulated MSCs were 3D cultured in a cell culture incubator (37°C, 5% CO_2_), and fresh cell culture medium (low-glucose DMEM component for cell proliferation evaluation while high-glucose DMEM component for cell differentiation evaluation) was replaced every 2 days.

### Evaluation of the encapsulated stem cell proliferation

The viability of MSCs encapsulated in SF hydrogels with varied conformational transition rates in the 12-well plate was assessed by the CTG 3D Cell Viability Assay (Promega) [22]. Firstly, the DMEM complete medium was removed from the wells containing the samples after 1, 4 and 7 days of cell culture. Then, 800 μL of a CTG and DMEM mixture (1:2) was added to each well. The plates were then incubated on a shaker at room temperature, protected from light, incubated for 1.5 h at 250 rpm. Finally, 150 μL of each reaction mixture was transferred into a brand-new 96-well plate, and the corresponding luminescence units (LU) to reflect the total cell viability were measured using a multimode plate reader (Tecan, Infinite 200). Then, the change ratio of total cell viability could be calculated and used to indirectly reflect the corresponding cell proliferation in the hydrogels.

### Live/dead staining and observation of the encapsulated stem cells

To more intuitively evaluate the growth and proliferation of encapsulated stem cells after 1 week of culture, three typical SF hydrogel groups (H-I, H-III and H-V) were selected and then subjected to live/dead staining. The procedure was as follows: Firstly, the encapsulated cells cultured in the 12-well plate were washed two times with 1× Assay Buffer (15 min for each time). Meanwhile, the related staining working solution was prepared by mixing the 1× Assay Buffer, Calcein-AM solution and PI solution at a volume ratio of 1000:1:3. Then, 1000 μl of the prepared staining working solution was added into each well and incubated for about 1.5 h in a cell culture incubator (37°C, 5% CO_2_). After that, the stained samples were further washed with 1× Assay Buffer (2 × 15 min). Finally, the fluorescence images were observed with the help of a laser confocal microscope (Zeiss, LSM700).

### Evaluation of the encapsulated stem cell chondrogenesis

#### Western-blot detection of chondrogenic-specific protein expression of MSCs

Typical chondrogenic-specific protein expressions of the stem cells within hydrogels of H-I ∼ H-Ⅴ were assessed to evaluate the influence of different conformational transition rates on cell chondrogenic differentiation.

After 6 and 12 days of culture, the samples were sent to Shanghai Daixuan Biotechnology Co., Ltd for western-blot (WB) detection, with the proteins of interest being ACAN (Aggrecan), Col II (Collagen type II alpha 1 chain), PRG4 (Proteoglycan 4) and COMP (Cartilage oligomeric matrix protein). The GAPDH (Glyceraldehyde-3-phosphate dehydrogenase) has been chosen as the reference protein. Briefly, the MSCs encapsulated in SF hydrogels after indicated culture were washed three times with pre-chilled PBS. RIPA lysis buffer was then added, and the samples were homogenized using a motorized homogenizer. The lysates were incubated on ice for 30 min, followed by centrifugation at 13 500 r/min for 15 min at 4°C. The supernatant, containing the extracted proteins, was collected and stored at −80°C for future use. Protein concentration was then measured according to the instructions provided with the BCA protein assay kit (DXWB010).

Then, the denatured protein samples were subjected to concentrated gel electrophoresis in electrophoresis buffer. After electrophoresis, the gel was soaked in transfer buffer along with a polyvinylidene fluoride (PVDF) membrane, and the protein was transferred from the gel to the PVDF membrane in an ice bath under oriented transfer conditions. The PVDF membrane, after protein transfer, was immersed in a 5% non-fat milk solution and incubated at room temperature for 1 h to block nonspecific binding. After the incubation, the blocking solution was discarded, and a primary antibody dilution (with a 1:1000 ratio of primary antibody to non-fat milk solution) corresponding to the target protein was added. The membrane was then incubated on a shaker at room temperature for 2 h. Following the incubation, the primary antibody solution was removed, and the PVDF membrane was washed three times with buffer (each for 10 min). Next, a secondary antibody dilution (with a 1:5000 ratio of secondary antibody to non-fat milk solution) was added, and the membrane was incubated at room temperature for 2 h. After the second incubation, the secondary antibody was removed, and the membrane was washed again three times with buffer (each for 10 min). Finally, the color reaction and chemiluminescence detection were performed. The molecular weight and relative expression levels of the target protein were determined by comparing the positions and intensities of the bands in different samples (H-I ∼ H-V).

#### RT-PCR detection of chondrogenic-specific gene expression of MSCs

Relative mRNA expression of chondrogenic markers of the stem cells within hydrogels (H-I ∼ H-V) was also detected to evaluate and confirm the influence of different conformational transition microenvironments on MSCs’ chondrogenic differentiation.

After 6 and 12 days of culture, the samples were sent to Shanghai Daixuan Biotechnology Co., Ltd for RT-PCR detection, with the typical genes of interest being ACAN, Col II, PRG4 and COMP. The GAPDH has been chosen as the housekeeping gene. Briefly, the MSCs encapsulated SF hydrogels were frozen and ground. Then, 50–100 mg of the hydrogel-cell complex was added to 1 mL of Trizol, and the mixture was gently pipetted to lyse the cells and release mRNA. Then, the mixture was thoroughly blended with chloroform at a volume ratio of 5:1 and centrifuged at 12 000 r/min for 15 min at 4°C. The upper aqueous phase was carefully transferred and mixed with an equal volume of isopropanol, followed by incubation at −20°C. The mRNA was then re-suspended in ethanol, followed by centrifugation and drying to obtain purified mRNA. Finally, the mRNA was dissolved in RNase-free water and used for the subsequent RT-PCR analysis.

After that, mRNA was reverse transcribed into cDNA using an M-MLV reverse transcriptase. Finally, RT-PCR was performed following the method described in previous literature [8, 17], with the relevant primer information for the genes listed in Table S3. The relative expression levels of ACAN, Col II, PRG4 and COMP genes in groups of H-I ∼ H-V were calculated by the 2^−ΔΔCt^ strategy.

### Statistical analysis

For each test conducted in this study, the parallel samples were at least three (*n *≥ 3, unless otherwise indicated), and all values were presented as the mean value ±SD. One-way analysis of variance (ANOVA) was used to assess the statistic difference between indicated samples, and it was considered to have significant statistical difference when *P *< 0.05.

## Results and discussions

This study aims to construct different SF hydrogels with highly similar initial properties while varied protein conformational transition rates, and further investigate the effects these conformational transition microenvironments on stem cell behaviors. So, we have first comprehensively investigated the initial properties and the corresponding transition processes of the fabricated hydrogels (H-I ∼ H-V). Then, the influence of different conformational transition rates on the proliferation and chondrogenic differentiation of encapsulated stem cells have been systematically revealed based on the fabricated material platform.

### The initial properties of fabricated SF hydrogels

Based on the previous discussion in the introduction, this study planned to prepare SF hydrogels with highly similar initial properties but with different conformational transition rates by altering the uniformity of crosslinking points while maintaining the similar crosslinking density. Herein, achieving highly similar crosslinking densities was a prerequisite for obtaining highly similar initial material properties. The exploration of the Ru/SPS and HRP/H_2_O_2_ quantities that result in SF hydrogels with highly similar compressive mechanical properties lays the foundation for establishing highly similar crosslinking density. Based on the prior investigations, it found that ‘11 quality units’ of HRP and ‘8.75 quality units’ of Ru can present highly similar initial compressive mechanical properties. Based on this valuable exploration, we further designed and constructed five kinds of SF hydrogels (H-I ∼ H-V) by adjusting the combination ratios of Ru and HRP according to their equivalent quality units. With the increase of HRP, the resulting SF hydrogels were sequentially named as H-I, H-II, H-III, H-IV and H-V.

**Figure 2.**
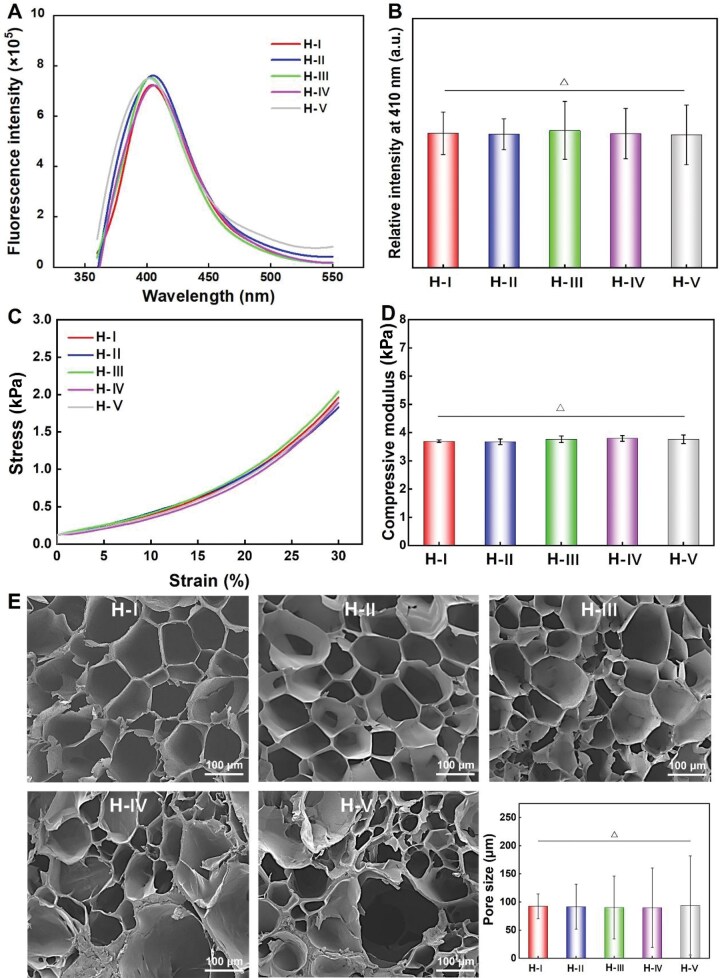
Initial properties evaluation of the fabricated SF hydrogels (H-I ∼ H-V). (**A**) fluorescence spectra of the SF hydrogels, (**B**) Relative fluorescence intensity of the hydrogels at the wavelength of 410 nm. (**C**) Stress–strain cures; (**D**) Compressive modulus; (**E**) Cross-sectional SEM images and the corresponding statistical results of the hydrogels pore size. ‘Δ’: *P *> 0.05.

Both the Ru/SPS and HRP/H_2_O_2_ crosslinking systems used to synthesize SF hydrogels rely on the formation of di-tyrosine bonds as the chemical crosslinking points of hydrogels [39]. Therefore, measuring the content of di-tyrosine bonds can directly reflect and confirm the hydrogel crosslinking density [34]. Figure 2A showed the characterized fluorescence spectra of the indicated hydrogels, which could be used to reflect the di-tyrosine bond contents of the hydrogels (H-I ∼ H-V), it indicated that the spectra are relatively similar between each other. Based on these spectra, we have also calculated and presented the statistical results of the fluorescence intensity at the characteristic peak of 410 nm [39], as shown in Figure 2B. It demonstrated that no significant difference could be found among these five kinds of hydrogels, which suggest that these fabricated hydrogels (H-I ∼ H-V) have highly similar initial crosslinking density.

At the same time, the initial mechanical properties of these hydrogels have also been detected. Typical compressive stress–strain curves of the fabricated SF hydrogels were illustrated in Figure 2C, where shows that the curves of the five hydrogels almost completely overlapped. Figure 2D showed the corresponding compressive modulus of each hydrogel, which are calculated from the data of Figure 2C. Specifically, the average compressive moduli of H-I ∼ H-V were 3.70 ± 0.05, 3.68 ± 0.10, 3.76 ± 0.12, 3.78 ± 0.10 and 3.70 ± 0.15 kPa, respectively. Statistical results revealed no significant differences in compressive moduli among the five SF hydrogels (*P *> 0.05), confirming that these hydrogels exhibit highly similar initial compressive mechanical properties.

Moreover, the important pore size features of these SF hydrogels have also been observed. Typical SEM images of these hydrogels’ cross-section views are shown in Figure 2E. The results indicated that H-I has a relatively uniform pore size feature, while the pore size heterogeneity of the SF hydrogels progressively increased from H-I to H-V (with the increasing of HRP contents in the hydrogel precursor). Further quantitative analysis of the pore sizes of H-I ∼ H-V was performed using Image J software. The statistical results (the column chart in the bottom right corner of Figure 2E) showed that the average pore sizes were 92.4 ± 22.1, 91.5 ± 40.3, 90.1 ± 55.8, 89.7 ± 70.6 and 93.7 ± 88.0 μm, respectively from H-I to H-V. On the one hand, the statistical *P*-values between each group were all greater than 0.05, suggesting highly similar initial average pore size exists in these SF hydrogels. On the other hand, the variance of the pore size data gradually increased from H-I to H-V, which indirectly indicated a progressive increase in the crosslinking point uniformity of these hydrogels.

In summary, the important initial properties such as crosslinking density, compressive mechanical property and average pore size of the fabricated SF hydrogels (H-I ∼ H-V) are all highly similar. The highly similar crosslinking density of these hydrogels is probably the main reasons for inducing similar mechanical property and average pore size among hydrogels of H-I ∼ H-V. In addition, during the synthesis of hydrogels from H-I to H-V, the amount of Ru gradually decreased, while the amount of HRP gradually increased. The molecular weight of HRP (Mw = 44 000 Da) is significantly larger than that of Ru (Mw = 750 Da). According to molecular dynamics and diffusion theory, small molecules diffuse more quickly and uniformly than larger molecules in the same medium due to their higher diffusion coefficients and weaker intermolecular forces [40, 41]. As a result, Ru, with its smaller molecular size, disperses more uniformly into the precursor system compared to the larger HRP, leading to a more homogeneous crosslinked hydrogel network. That’s probably why the pore size heterogeneity (indirectly indicating the crosslinking point uniformity of SF hydrogels) increased from H-I to H-V.

### The process of conformational transition within the fabricated hydrogels

**Figure 3.**
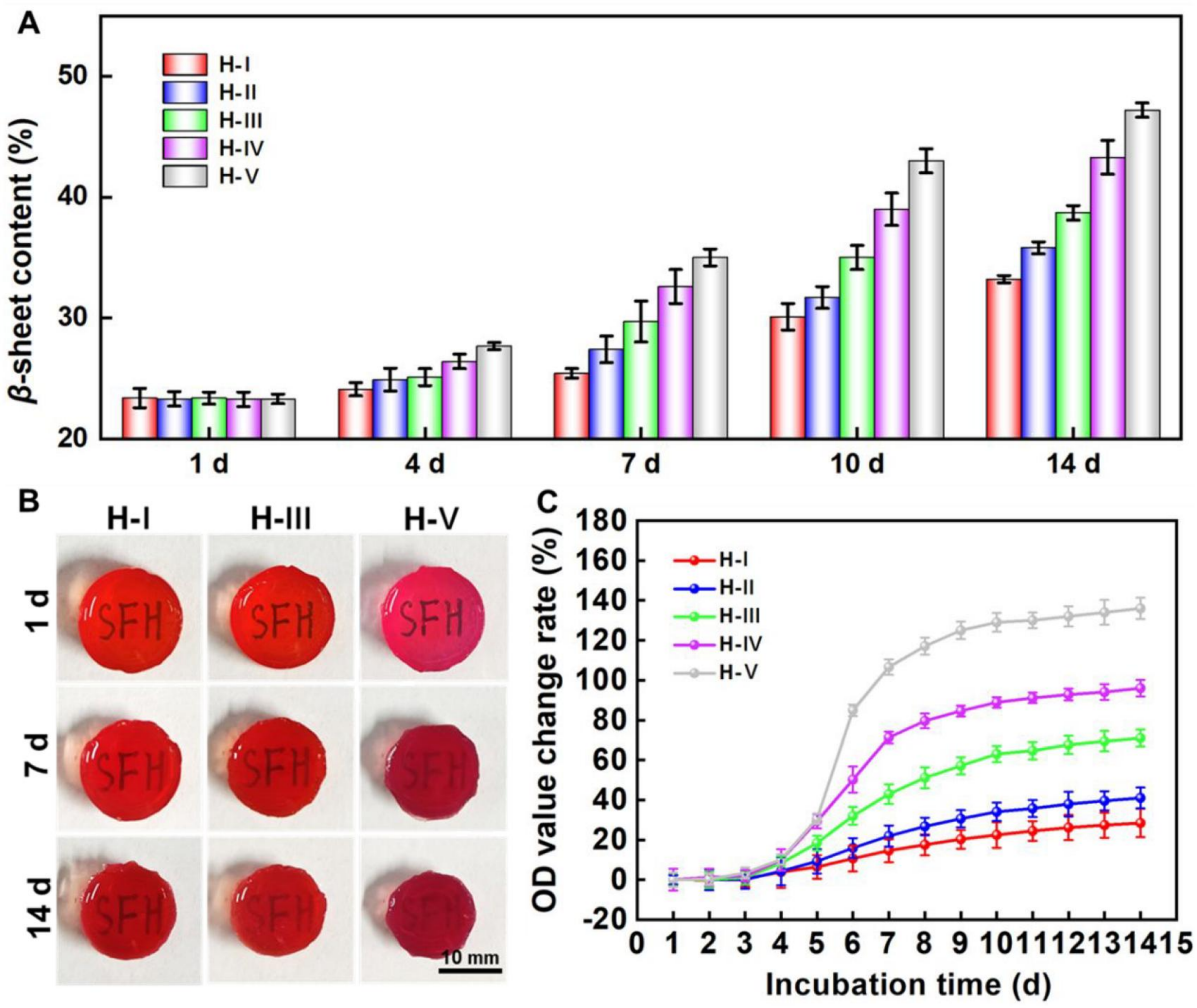
Evaluation of the process of conformational transition within the fabricated hydrogels. (**A**) The changes of the *β*-sheet structure contents within SF hydrogels after indicated incubation times; (**B**) The changes of the macroscopic states of typical hydrogels after indicated incubation times; (**C**) The changes of the OD values at 620 nm of SF hydrogels after indicated incubation times.

The mentioned conformational transition refers to the process in which protein chain segments shift from a random coil to a *β*-sheet conformation [23]. Therefore, monitoring the changes of *β*-sheet conformation content could provide direct evidence of the protein conformational transition in the designed hydrogels. The corresponding hydrogels after 1, 4, 7, 10 and 14 days of incubation were freeze-dried and then characterized by FT-IR accordingly [37]. Then, the specific *β*-sheet contents in SF hydrogels along with incubation times were calculated and shown in Figure 3A. Initially, the *β*-sheet contents in all groups were approximately 23.3%, with no significant differences between different SF hydrogels. With the extension of incubation time, the contents of *β*-sheet structure within all groups gradually increased, while the related increasing rates were different. Specifically, after 7 days of incubation, the *β*-sheet content in H-I ∼ H-V increased to 25.3%, 27.4%, 29.7%, 32.6% and 35.3%, respectively; after 14 days, it further increased to 33.3%, 35.8%, 38.7%, 43.4% and 47.2%, respectively. Overall, the corresponding transition rate is gradually increased in the order as H-I, H-II, H-III, H-IV and H-V, which is inversely related to the uniformity of crosslinking points.

It has been reported that the conformational transition will decrease the hydrogel transparency gradually. Therefore, we have also captured three typical hydrogels’ gross views to more intuitively illustrate the process of the conformational transition within the indicated hydrogels (Figure 3B). It also proved that the corresponding transition rate is gradually increased in the order as H-I, H-III and H-V. In addition, the trends of OD value change rate at 620 nm during the transition process have also been carefully detected to quantitatively characterize the transparency changes of the SF hydrogels. Results (Figure 3C) showed that the change rates were quite different among the five kinds of hydrogels. For example, after 4 days of incubation, the OD value changes rates from H-I, H-II, H-III, H-IV to H-V were 3.8%, 4.3%, 8.2%, 9.9% and 10.2%, respectively; after 10 days of incubation, the OD value changes rates were 22.4%, 34.0%, 63.0%, 88.9% and 129%, respectively; after 14 days of incubation, the OD value changes rates become 28.4%, 40.9%, 71.1%, 96% and 136%, respectively. Summarily, the order of transparency changes rates of SF hydrogels from slow to fast is also H-I, H-II, H-III, H-IV and H-V. This is in complete agreement with the change trend of *β*-sheet structure contents of the indicated hydrogels (Figure 3A), further confirming the varied conformational transition microenvironments within the fabricated hydrogels.

As we know, natural SF molecules possess a unique structure composed of alternating hydrophilic and hydrophobic chain segments. And the hydrophobic segments could easily self-assemble with each other to form *β*-sheet crystalline structures. The inherent hydrophobic and hydrophilic chain arrangement enables spontaneous formation of regularly alternating *β*-sheet crystalline domains and amorphous domains during the natural spinning process [4, 42]. This distinctive organized hierarchical architecture endows natural silk with excellent mechanical strength and toughness. In this report, the fabricated SF hydrogels are formed through crosslinking between tyrosine in their hydrophilic molecular segments. Consequently, SF molecules in these hydrogels still possess ‘intact’ hydrophobic chain segments. Based on the intrinsic characteristics of natural SF molecules, the free movement and self-assembly of hydrophobic chain segments inevitably induce a conformational transition of the SF molecules from random coil structures to *β*-sheet structures with lower-energy state [14, 23]. This constitutes the fundamental mechanism underlying protein conformational transitions within the fabricated SF hydrogels.

The above-mentioned results proved that the SF hydrogels with high uniform crosslinking points exhibited slower conformational transition, while those with low uniform crosslinking points underwent faster conformational transition. Under similar crosslinking density, the hydrogel with LU of crosslinking points would exist some relative ‘longer’ hydrophobic molecular segments compared to the hydrogel owning HU of crosslinking points. The ‘longer’ hydrophobic segments in the hydrogel with LU of crosslinking points are more likely to move freely and self-assemble [14, 33], thereby inducing faster protein conformational transitions. In addition, as the increasing of *β*-sheet contents, the transparency ability and volume of the SF hydrogel decreased [14, 22, 23]. That’s probably why the hydrogels with faster conformational transition also present faster transparency decreasing (Figure 3B and C) and size contraction trends (Figure 3B).

In summary, these findings proved that the regulation of conformational transition microenvironments of SF hydrogels could be achieved by changing the uniformity of crosslinking points. Based on the developed material strategy, five kinds of SF hydrogels (H-I ∼ H-V) with highly similar initial properties while different conformational transition rates have been successfully fabricated.

### Effects of the varied protein conformational transition processes on the pore characteristics of the hydrogels

**Figure 4.**
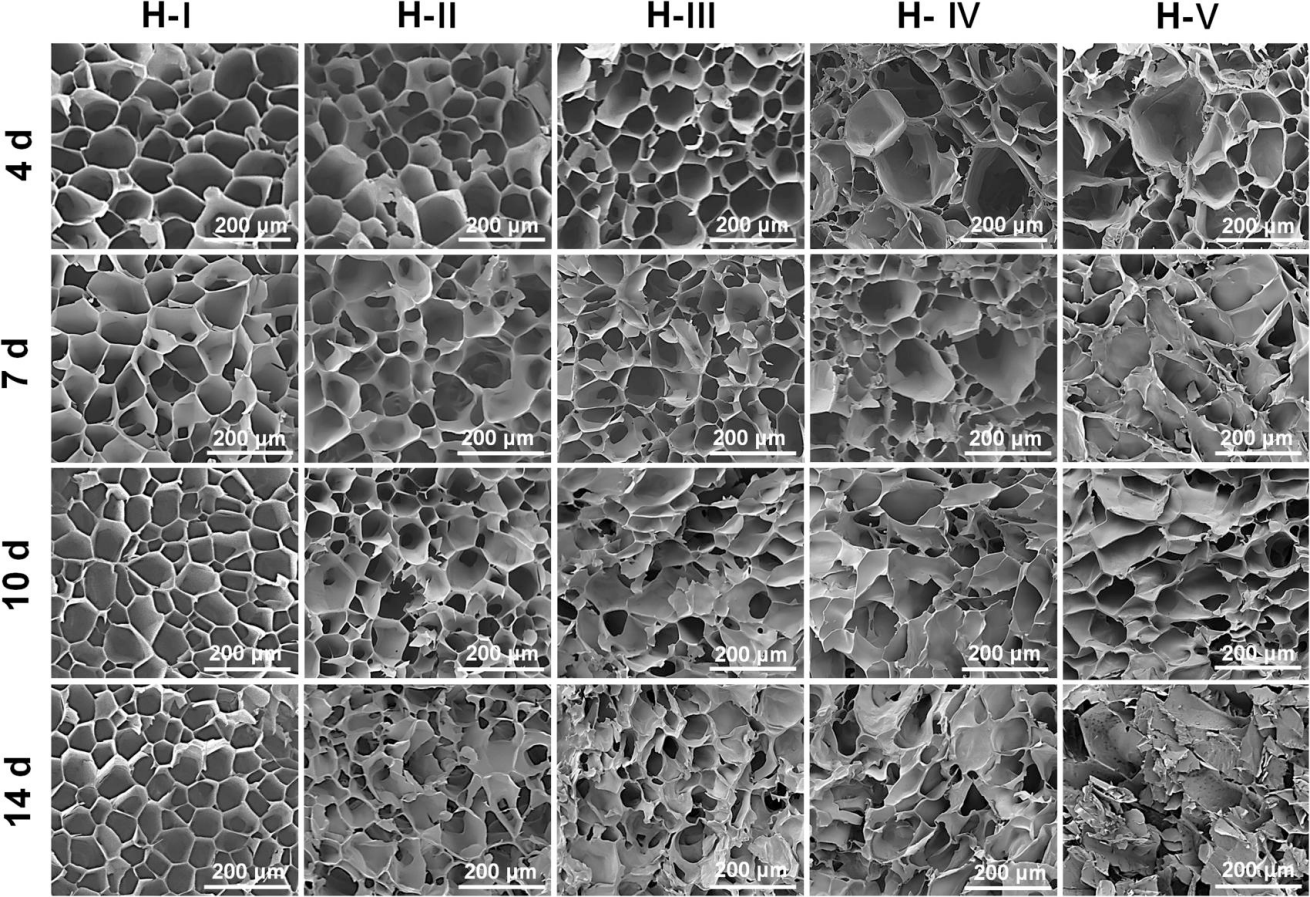
SEM Photographs of the pore structures within indicated hydrogels during the corresponding conformational transition processes. The SEM photographs of the initial state could be seen in Figure 2E.

The mentioned protein conformational transition could deeply affect the internal pore structures of the hydrogels. So, we have carefully characterized the structural features of the hydrogel cross-section during the corresponding transition process. Typical SEM photographs of the pore structures within SF hydrogels during the protein conformational transition processes were shown in Figure 4.

There were no obvious statistical differences among the average pore sizes of H-I ∼ H-V at the initial state (as mentioned and discussed in section The Initial Properties of Fabricated SF Hydrogels, Figure 2E). After 4 days of incubation, the pore sizes have slightly decreased, probably because of the slight conformational transition and weak contraction during the early incubation stage. With the extension of incubation time (about 1 week), the pore sizes feature of H-III, H-IV and H-V has obviously decreased compared to their initial state, especially the groups of H-IV and H-V own the fastest conformational transition processes. Furthermore, even the groups with the slowest conformational transition processes (H-I and H-II) showed significant pore size decreasing compared to their initial state when after 10 days to 2 weeks of incubation. As for the pore size decreasing along with protein conformational transition, it probably owing to that the increased *β*-sheet structures within SF hydrogels (Figure 3A) increased the physical crosslinking density and decreased the volume of hydrogels (Figure 3B). At this later stage, some obvious pore structure collapse has also happened on the groups with faster conformational transition rates (H-IV and H-V), mainly because of the faster material contraction and the lowest uniformity of the crosslinking points and pore structures.

Summarily, these results proved that the mentioned transition process of protein conformation in SF hydrogel would induce material contraction and pore size decreasing effects. And the micromorphology feature change rate and degree are both positively correlated with the corresponding transition rate of protein conformation within these hydrogels.

### Effects of the varied protein conformational transition processes on the compressive mechanical characteristics of the hydrogels

Apart from pore size, the compressive mechanical properties are also important characteristic of SF hydrogels. These material cues could all have profound effects on the related cell behaviors. Therefore, the effects of varied conformational transition rates on the mechanical properties of SF hydrogels have also been carefully investigated in this report.

**Figure 5.**
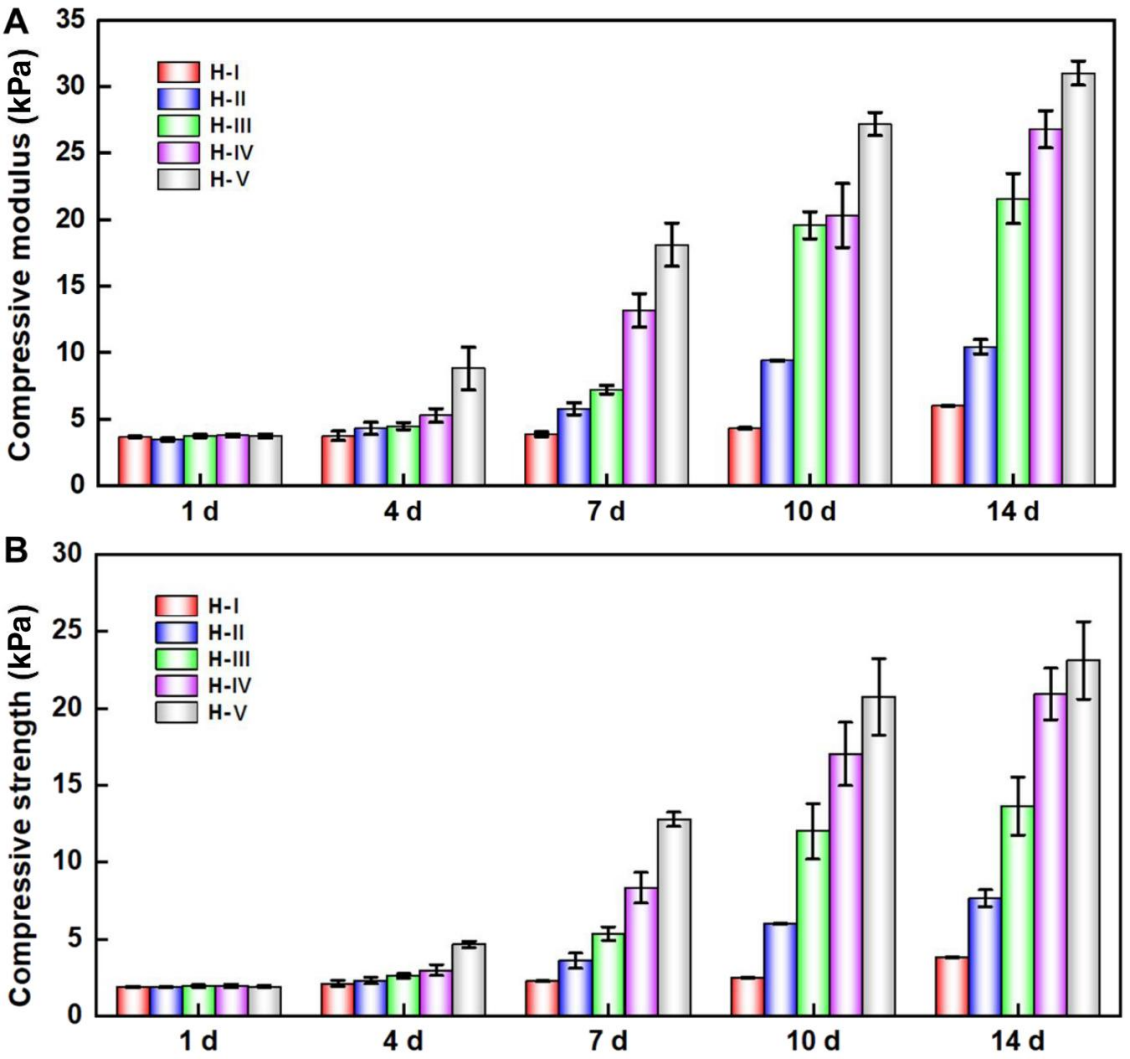
Compressive mechanical property changes of the different SF hydrogels during the corresponding conformational transition processes. (**A**) The changing of compressive modulus; (**B**) The changing of compressive strength.

The tested stress–strain curves of H-I ∼ H-V using compression mode at different hydrogel incubation time points were shown in Figure S2. In order to better reflect the change trends of the material compressive mechanical properties, we have calculated the corresponding modulus and strength of these hydrogels from the corresponding stress–strain data and separately presented them in Figure 5A and B. At the initial state, the compressive moduli of H-I ∼ H-V were 3.70, 3.68, 3.76, 3.78 and 3.70 kPa, respectively, and the compressive strengths were 1.90, 1.89, 1.93, 1.92 and 1.90 kPa, respectively. There were no statistically significant differences among each group in both the compressive modulus and strength. That’s probably because the hydrogels of H-I ∼ H-V have highly similar crosslinking density. As the incubation time increases, the mentioned modulus of all the SF hydrogels gradually elevated, and the increasing rate order from low to high is H-I, H-II, H-III, H-IV and H-V. As an example, after 1 week incubation, the compressive moduli of H-I and H-II were slightly increased to 3.90 and 5.80 kPa, and those of H-III, H-IV and H-V were significantly increased to 7.23, 13.21 and 18.13 kPa, respectively. After 2 weeks incubation, the compressive moduli of H-I and H-Ⅱ were increased to 6.03 and 10.47 kPa, and those of H-III, H-IV and H-V were further obviously increased to 21.61, 26.83 and 31.03 kPa, respectively. Similar increasing rate order (from low to high: H-I, H-II, H-III, H-IV and H-V) has also been found in the compressive strength. For example, after 1 week incubation, the compressive strengths of H-I and H-II were slightly increased to 2.27 and 3.60 kPa, and those of H-III, H-IV and H-V were significantly increased to 5.36, 8.35 and 12.80 kPa, respectively. After 2 weeks incubation, the compressive strengths of H-I and H-II were increased to 3.81 and 7.66 kPa, and those of H-III, H-IV and H-V were further obviously increased to 13.65, 20.94 and 23.11 kPa, respectively.

During the protein conformational transition process, the *β*-sheet structure contents within SF hydrogels gradually increased, thus condensing the structure and enhancing the mechanical properties of hydrogels. So, the compressive modulus and strength increasing rates order of SF hydrogels from slow to fast is H-I, H-II, H-III, H-IV and H-V (Figure 5), which is positively correlated with the corresponding transition rates of protein conformation within these hydrogels.

### Effects of the varied protein conformational transition microenvironments on the proliferation of stem cells within hydrogels

The above results have demonstrated that the fabricated SF hydrogels of H-I ∼ H-V have highly similar initial properties while different conformational transition rates, which have provided a powerful material platform for revealing the influence of conformational transition microenvironment within hydrogels on the internally cultured stem cell behaviors. Therefore, we further chose MSCs as a typical cell to reveal the effects of varied conformational transition processes within SF hydrogels on the proliferation and chondrogenic differentiation of encapsulated stem cells.

**Figure 6.**
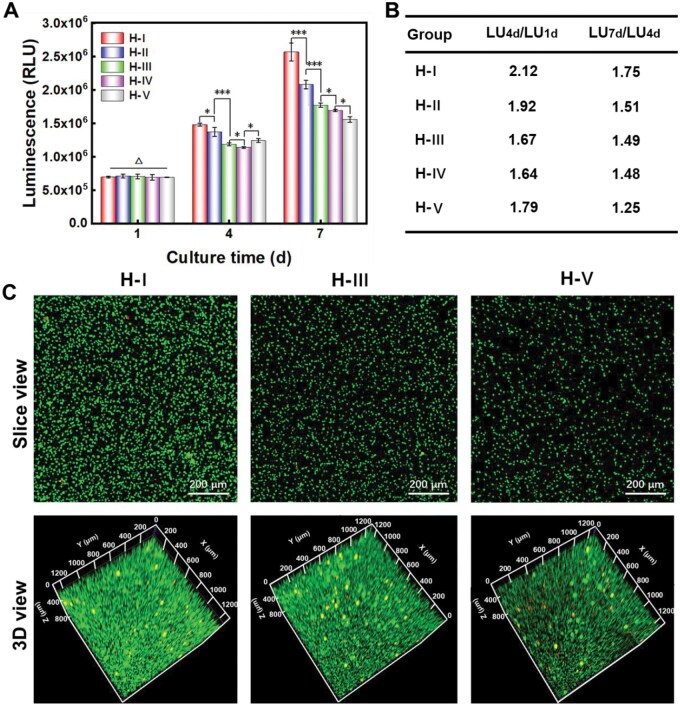
Effects of the varied protein conformational transition microenvironments on the proliferation of stem cells within hydrogels. (**A**) Relative total cell viability changes during the corresponding conformational transition processes; (**B**) Related change ratios of the LU values from the data of (A); (**C**) LSCM photographs of the live/dead staining of the MSCs within typical hydrogels after 1 week cell culture. ‘Δ’: *P *> 0.05; ‘*’: 0.01 < *P *< 0.05; ‘**’: 0.001 < *P *< 0.01; ‘***’: *P *< 0.001. The calculated *P* values for evaluating the statistical difference between indicated groups in (A) are presented in Tables S4–S6.

As for cell proliferation evaluation, the relative viabilities of MSCs encapsulated in H-I ∼ H-V were assessed after 1, 4 and 7 days of cell culture (Figure 6A). The total cell viability could be used to reflect the total cell numbers, thus indirectly reflect cell proliferation. Initially, there was no statistical difference in the cell viabilities among H-I ∼ H-V. After 4–7 days of cell culture, the total cell viability increased different extents among different groups. Based on these LU values, we calculated the LU value change rates to more intuitively reflect the cell proliferation rate during the indicated culture period (Figure 6B). The LU_4d_/LU_1d_ values showed that cells encapsulated in H-I presented the fastest cell proliferation during the period of 1 ∼ 4 days. Except for H-V, the cell proliferation speed showed a gradually decreasing trend from H-I to H-IV during this initial period. In addition, during the period of 4 ∼ 7 days, the LU_7d_/LU_4d_ ratios illustrated that stem cells encapsulated in H-I still have the best cell proliferation. And it showed a monotonically decreasing proliferation rate from H-I ∼ H-V during this later period. To visually demonstrate the total cell numbers after 7 days of culture, live (green)/dead (red) staining was also performed in the typical hydrogels of H-I, H-III and H-V (Figure 6C). Notably, both slice view and 3D view indicated that H-I had the maximum live cell numbers, while H-V exhibited the minimum live cell numbers, which were well consistent with the proliferation trend obtained by CTG test.

It has been reported that the pore size of hydrogels is an important factor for influencing 3D cultured cell proliferation [43, 44]. More specifically, relatively larger pore sizes could be beneficial for cell proliferation [43, 45]. On the one hand, larger pore size could afford sufficient and suitable space for cell growth. On the other hand, it also facilitates the exchange of nutrients and metabolic waste [44]. Recently, some classic literature have also reported that the dynamic mechanical stimuli [17, 30] and other dynamic material features [10, 46] could effectively influence the encapsulated cell proliferation within cell culture substrate. For example, it has been reported that suitable material stiffening process could enhance cell proliferation [14, 47, 48].

In this study, the mechanical properties and average pore sizes of H-I∼H-V were initially highly similar, resulting in similar MSCs viability at the initial time point. After 4 days of culture, the conformational transition in the SF hydrogels increased their compressive moduli, while the increase degree of all the hydrogels was small (Figure 5A). So, the main factors for influencing cell proliferation might be pore size during this period. The mentioned conformational transition during 1–4 days led to a reduction in pore size, with a greater reduction degree in the hydrogels with faster conformational transition. Therefore, the encapsulated cell proliferation rate should be decreased with the increasing of conformational transition rate. The data in Figure 6B (LU_4d_/LU_1d_) for evaluating cell proliferation during 1–4 days basically conform to this rule except for the group of H-V. It showed that the MSCs in H-V exhibited higher cell proliferation rate than those in H-III and H-IV. That’s probably because the pore size distribution of H-V is wider than H-III and H-IV, thus some ‘large sized’ pores exist in this hydrogel (Figure 4). Although there is a more obvious pore size decreasing trend in H-V, the pore structure still remains intact within the initial 4 days, and these decreased ‘large sized’ pores could still better support cell proliferation than H-III and H-IV. In addition, the more obvious and suitable material stiffening process of H-V during the initial 4 days (Figure 5) might also be more favorable for cell proliferation than H-III and H-IV. After 7 days of culture, the pore size further decreased, and parts of the ‘large sized’ pores in the H-V would be collapsed (Figure 4), so the mentioned advantages of the ‘large sized’ pores no longer exist at this later period. In addition, the excessively small pore size in H-V induced unfavorable cell growth could also conceal the possible enhancing effects of the dynamic material stiffening on cell proliferation during this period. Therefore, the encapsulated cell proliferation rates were decreased as the increasing of conformational transition rate. The data in Figure 6B for evaluation cell proliferation (LU_7d_/LU_4d_) could well match this rule.

When eliminating the possible interference of the varied initial hydrogel features as much as possible, the current report has proved that the SF hydrogel with slower conformational transition rate was more favorable for stem cell proliferation. Accompanied by different modulus and pore size features, our previous report [14] has illustrated that the hydrogel with moderate conformational transition rate was more suitable for cell proliferation. The different cell response trends could probably be due to the important cues for effecting cell proliferation in the previous report [14] not only contained the varied dynamical protein conformational transition, but also the different initial hydrogel modulus and pore sizes induced by different hydrogel crosslinking densities. Summarily, the conformational transition microenvironment within SF hydrogels could effectively regulate encapsulated cell proliferation. All the hydrogels could well support cell proliferation and the group with slower conformational transition rate was more favorable for cell proliferation when eliminating the possible interference of the varied initial hydrogel characteristics as much as possible. The phenomenon was probably due to the integrated effects of pore structure shrinkage and material stiffening process caused by the varied conformational transition microenvironments.

### Effects of the varied protein conformational transition microenvironments on the chondrogenic differentiation of stem cells within hydrogels

SF hydrogels exhibit significant potential for applications in the cartilage repairing field [8, 17, 49–51]. Based on this application scenario, we further carefully investigated the chondrogenic differentiation of MSCs within these hydrogels.

**Figure 7.**
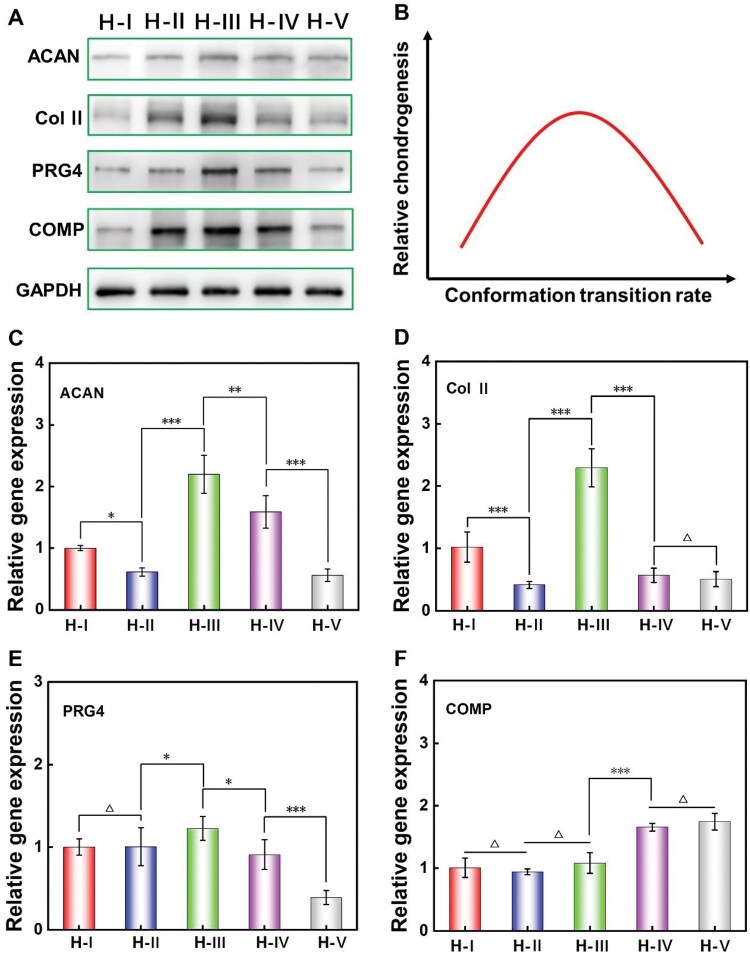
Effects of the varied protein conformational transition microenvironments on the chondrogenic differentiation of stem cells within hydrogels after 6 days of culture. (**A**) WB results of the ACAN, col II, PRG4 and COMP; (**B**) Schematic diagram to illustrate the relative comprehensive chondrogenesis trend along with the conformational transition rate; (**C–F**) Relative chondrogenic characteristic genes expression of ACAN, col II, PRG4 and COMP, respectively. ‘Δ’: *P *> 0.05; ‘*’: 0.01 < *P *< 0.05; ‘**’: 0.001 < *P *< 0.01; ‘***’: *P *< 0.001. The calculated *P* values for evaluating the statistical difference between indicated groups in (C–F) are presented in Tables S7–S10.

To directly analyze the chondrogenesis ability of MSCs encapsulated in H-I∼H-V, WB was performed on Day 6 to detect the expression of cartilage-specific proteins. The expression levels of cartilage-specific proteins ACAN, Col II, PRG4 and COMP in H-I∼H-V were shown in Figure 7A. The relative intensity of band coloration in the immunoassay exhibited a positive correlation with the expression levels of the indicated proteins, wherein darker bands reflect higher protein expressions [52, 53]. Comprehensively, it illustrated that the highest cartilage-specific markers expression happened on MSCs encapsulated in H-III. In addition, the expression of these specific markers exhibited a medium level in H-II and H-IV, and lowest level in H-I and H-V. Based on the data from Figure 7A and the above analysis, a schematic diagram to illustrate the relative chondrogenic differentiation capability of MSCs encapsulated in H-I∼H-V was drawn (Figure 7B). It clearly indicated that the chondrogenesis capability of MSCs increased initially and then decreased as the conformational transition rates accelerated, and the peak value appeared at H-III with medium conformational transition rate.

To more comprehensively reflect the chondrogenic differentiation status, this study also utilized RT-PCR to measure the mRNA expression levels of the cartilage-specific genes at Day 6, related results were shown in Figure 7C–F. Comprehensively, except for the COMP, the maximum expression of ACAN, Col II and PRG4 were all happened on the H-III. In addition, most of the specific gene expression was higher in H-IV when compared with H-V. These results were well consistent with the chondrogenesis trends obtained from WB results (Figure 7B). As for H-I and H-II, the comprehensive genes expression showed a little higher level on H-I when compared with H-II. Theoretically, the RT-PCR data only reflects the transient (a relatively short period) expression levels of genes in cells under related conditions, and therefore exists a certain degree of random volatility. That’s probably why the COMP gene expression and the relationship between H-I and H-II here (Figure 7C–F) presented ‘undesirable’ trends when compared to the WB results (cumulative expression level of specific proteins, Figure 7B).

In 3D cell culture system, the cell density, material pore size features, and even some dynamical material cues all played crucial roles in regulating the chondrogenic differentiation of encapsulated stem cells. More specifically, typical literatures have reported that high cell density and cell aggregation were beneficial for cell chondrogenesis [54, 55]. As for the material pore sizes, proper medium-sized pores are favorable for cell chondrogenesis, while too large or too small pores were not so suitable [56–58]. That’s probably because excessively small pore size could induce insufficient nutrient supply and obstruct cell function. Excessively large pore size would hinder cell aggregation and reduce cell–cell contacts, thus also weakening cell chondrogenesis. Besides the mentioned static material cues, some frontier reports have also demonstrated that dynamical material cues such as suitable material degradation [10] and material stiffening processes [14, 23, 47] could have significant enhancing impacts on stem cell chondrogenesis.

At the early cell culture stage (6 days), although the total cell numbers in H-III with medium conformational transition are less than H-I and H-II (Figure 6A), the properly reduced pore structure (shrinkage) and suitable dynamical stiffening process induced by medium conformational transition rate might be more beneficial for cell chondrogenesis, thus inducing the highest cell chondrogenesis ability in H-III. As for H-I, although the total cell number was the highest (Figure 6A), no obvious stiffening/shrinkage process happened in this hydrogel with the slowest conformational transition rate (Figure 5), thus comprehensively inducing a weaker cell chondrogenesis than H-II. As for the H-V with the fastest conformational transition rate, the fast-dynamical material stiffening process might be useful for chondrogenesis, while the smallest average pore size restricted cell proliferation (Figure 6B) and obstructed the functioning of cells, thus also comprehensively inducing the weakest cell chondrogenesis. In summary, during the period of 1 ∼ 6 days, the chondrogenesis of stem cells within hydrogels (H-I ∼ H-V) were mainly influenced by the pore size and the dynamical material stiffening cues induced by the corresponding conformational transition, and the hydrogel with a moderate transition rate of protein conformation could obviously accelerate stem cell chondrogenesis within hydrogels.

**Figure 8.**
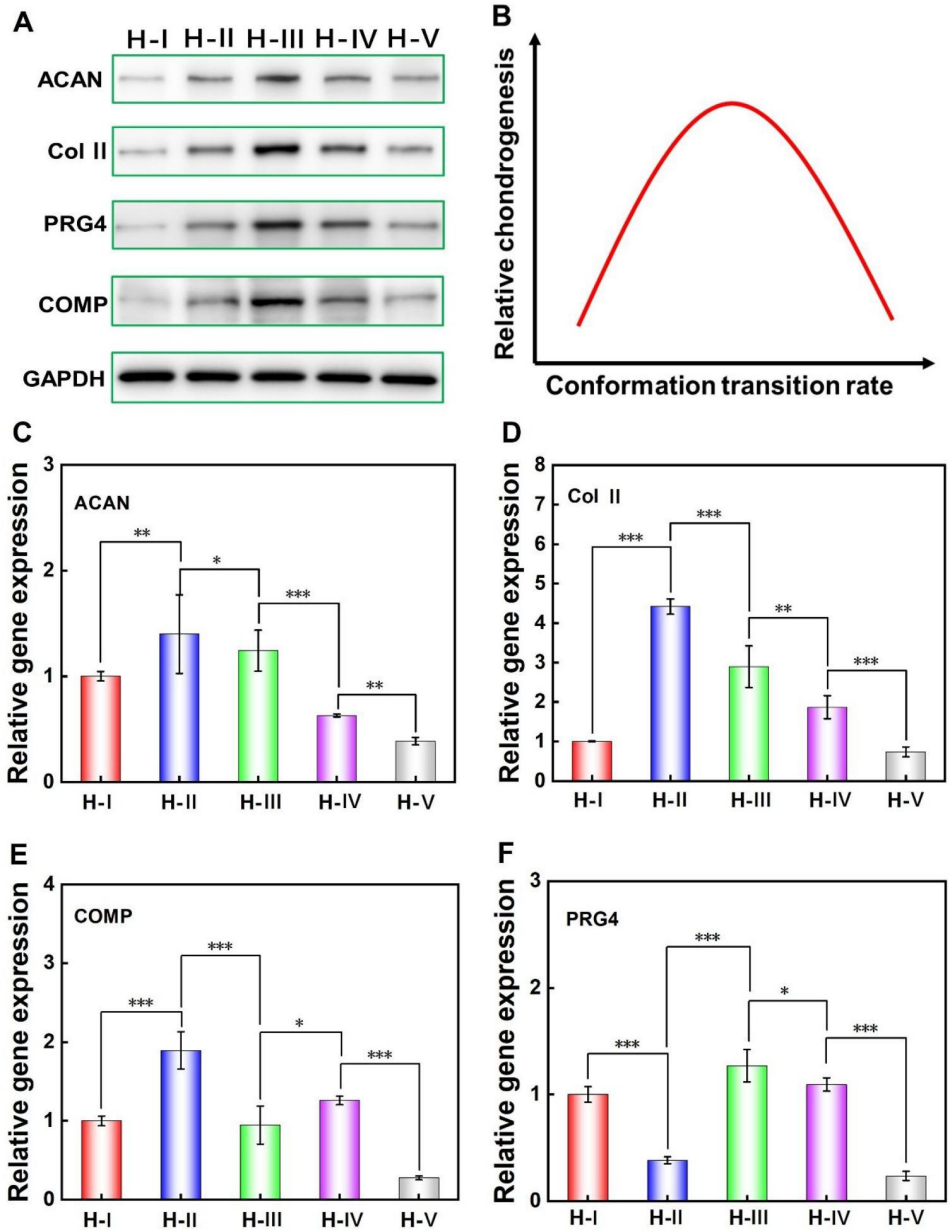
Effects of the varied protein conformational transition microenvironments on the chondrogenic differentiation of stem cells within hydrogels after 12 days of culture. (**A**) WB results of the ACAN, col II, PRG4 and COMP; (**B**) Schematic diagram to illustrate the relative comprehensive chondrogenesis trend along with the conformational transition rate; (**C–F**) Relative chondrogenic characteristic genes expression of ACAN, col II, COMP and PRG4, respectively. ‘*’: 0.01 < *P *< 0.05; ‘**’: 0.001 < *P *< 0.01; ‘***’: *P *< 0.001. The calculated *P* values for evaluating the statistical difference between indicated groups in (C–F) are presented in Tables S11–S14.

We further investigated the cell chondrogenesis situation after 12 days of culture, the expression levels of cartilage-specific proteins were analyzed and shown in Figure 8A, which illustrated a quite similar trend as that obtained after 6 days of culture (Figure 7A). MSCs encapsulated in H-III exhibited the highest expression of ACAN, PRG4, Col II and COMP proteins, and the protein expression levels gradually decreased from H-III to H-V and H-III to H-I, as comprehensively and schematically illustrated in Figure 8B.

Meanwhile, the RT-PCR testing was conducted and shown in Figure 8C–F. Except for PRG4, the maximum gene expression of ACAN, Col II and COMP was all happened on the H-II. In addition, most of the specific gene expression from high to low is H-II, H-III, H-IV, H-I and H-V. Compared with the chondrogenesis trend obtained by the WB results (Figure 8B), the biggest difference lies in the shifting of the highest peak value from H-III in the WB results to H-II in the RT-PCR results. As mentioned before, being different from WB data, the RT-PCR data only reflect the transient expression levels of genes in cells during a relatively short period. Therefore, the revealed peak value difference at Day 12 might hint that the chondrogenic ability of cells in H-II was higher than that of the H-III at the very late stage of this period. Because the WB results reflect the cumulative expression level of indicated proteins during 1–12 days, they still presented the highest expression in the H-III, just the same as that presented at Day 6, which is the intermediate time point.

The gene expression illustrated that H-II has the ‘transit’ optimal chondrogenic induction ability at the very late stage of 1–12 days (shift from H-III at Day 6 to H-II at Day 12). The possible reasons are as follows: (1) During the mentioned later stage, the relatively slow conformational transition rate of H-II makes its pore size decrease to a suitable range which is favorable for cell chondrogenesis; (2) Since the dynamical material stiffening cues of H-II induced by the corresponding conformational transition at the later stage were quite similar to those of H-III during the medium stage, this suitable dynamical stiffening cue could further elevate stem cell chondrogenesis. As for the H-IV and H-V with faster conformational transition rate, the excessively fast dynamical stiffening process induced by faster conformational transition might be unfavorable for cell chondrogenesis, and the induced excessively small pore size could obviously further restrict cell proliferation and functioning, thus comprehensively causing the weakest cell chondrogenesis. As for H-I with the slowest conformational transition rate, the relatively large pore size (Figure 4) and weakest stiffening process (Figure 5) were not beneficial for cell chondrogenesis, thus also inducing weaker cell chondrogenesis.

In the related field, the reported influence of the conformational transition of SF-based hydrogels coupled with different initial hydrogel features on cell differentiation has also hinted that moderate conformational transition could promote the chondrogenesis of stem cells [14, 23]. Compared with them, the current report has accurately confirmed that the moderate microenvironment of material stiffening/shrinkage induced by appropriate conformational transition exerts significant enhancing effects on stem cell chondrogenesis, as it minimizes the possible interference of the varied initial hydrogel features as much as possible. Together, as for cell chondrogenesis, this report could further hint that these novel dynamical material cues induced by conformational transition may be much stronger than other static material cues, such as the chemical composition [23] and initial modulus [14]. This may also explain why optimal chondrogenesis consistently occurred on SF-based hydrogels with moderate conformational transition rates, even when using different material platform construction strategies [14, 23].

In summary, the stem cell chondrogenesis within SF hydrogels could be effectively regulated by the corresponding protein conformational transition microenvironment. Comprehensively, the hydrogel with moderate transition rate of protein conformation could obviously improve the encapsulated stem cell chondrogenesis when compared to those with slow or fast conformational transition rates. Except for the material pore size, the dynamical material cues such as dynamical stiffening/shrinkage induced by the conformational transition have afforded another important cue for regulating 3D cultured cell behaviors.

## Conclusions

This study successfully developed a series of SF hydrogels with highly similar initial properties but different conformational transition rates by adjusting the ratios between the two crosslinking triggering systems (Ru/SPS and HRP/H_2_O_2_). Results showed that these hydrogels exhibited highly similar crosslinking density, initial mechanical and average pore size features. Under these highly similar initial characteristics, SF hydrogels with higher uniformity of crosslinking points exhibited lower conformational transition rate, and *vice versa*. Moreover, accelerated conformational transition rates in SF hydrogels correlate with higher rates of compressive modulus increase and pore size reduction, thereby promoting rapid dynamic stiffening and shrinkage of the material microenvironment. Based on this powerful material platform, this study further systematically investigated and precisely revealed the effects of the conformational transition microenvironments on the proliferation and chondrogenic differentiation of stem cells within SF hydrogels. Cell responses further clearly confirmed that the protein conformational transition microenvironment could obviously influence cell behaviors. More specifically, a slower conformational transition rate significantly enhanced encapsulated-stem-cell proliferation, whereas a moderate transition rate promoted encapsulated-stem-cell chondrogenesis. Through powerful material construction techniques, this study revealed the unknown effects of the dynamical material microenvironment of protein conformational transition on stem cell behaviors within SF hydrogels. This finding not only deepens our understanding of cell-material interactions, but also provides critical insights for designing effective SF-based cell culture matrix and tissue engineering scaffolds.

## Supplementary Material

rbaf102_Supplementary_Data
