# Supplementary material for: Protein conformational transition microenvironment in silk fibroin hydrogels: proliferation and chondrogenesis of encapsulated stem cells
Source: Regen Biomater. 2025 Oct 1;12:rbaf102. doi: 10.1093/rb/rbaf102 (PMC12598285; doi:10.1093/rb/rbaf102)
Supplement: rbaf102_Supplementary_Data [file rbaf102_supplementary_data.zip › Conformational transition--SI--20250817.docx]

Supporting information for

**Protein conformational transition microenvironment in silk fibroin hydrogels: Proliferation and chondrogenesis of encapsulated stem cells**

Weikun Zhao, Guolong Cai, Jiayao Qian, Jingjing Geng, Xiang Yao*, Yaopeng Zhang*

State Key Laboratory of Advanced Fiber Materials, College of Materials Science and Engineering, Shanghai Engineering Research Center of Nano-Biomaterials and Regenerative Medicine, Donghua University, Shanghai, 201620, P. R. China

*Correspondence address. E-mail: yaoxiang@dhu.edu.cn (X.Y.); zyp@dhu.edu.cn (Y.Z.)

**Table S1** The formulation of precursor solutions for various kinds of HRP and Ru SF hydrogels

| Sample code | *V*(SF)/mL | *V*(Ru)/ μL | *V*(SPS)/μL | *V*(HRP)/ μL | *V*(H_2_O_2_)/μL |
| --- | --- | --- | --- | --- | --- |
| Ru35 | 2 | 35  (0.85×10^-3^ mol/L) | 35  (0.85×10^-2^ mol/L) | / | / |
| Ru45 | 2 | 45  (1.07×10^-3^ mol/L) | 45  (1.07×10^-2^ mol/L) | / | / |
| Ru55 | 2 | 55  (1.31×10^-3^ mol/L) | 55  (1.31×10^-2^ mol/L) | / | / |
| HRP40 | 2 | / | / | 40  (19.41 U/mL) | 20  (0.48×10^-2^ mol/L) |
| HRP50 | 2 | / | / | 50  (24.10 U/mL) | 25  (0.59×10^-2^ mol/L) |
| HRP60 | 2 | / | / | 60  (28.71 U/mL) | 30  (0.70×10^-2^ mol/L) |
| HRP44 | 2 | / | / | 44  (21.30 U/mL) | 22  (0.52×10^-2^ mol/L) |
| HRP48 | 2 | / | / | 48  (23.17 U/mL) | 24  (0.57×10^-2^ mol/L) |

Notes：The concentration of SF solution is 3 wt%; the initial concentration of HRP solution is 1000 U/mL；the initial concentration of H_2_O_2_ is 0.49M；the initial concentration of Ru is 0.05M；the initial concentration of SPS is 0.5M.

**
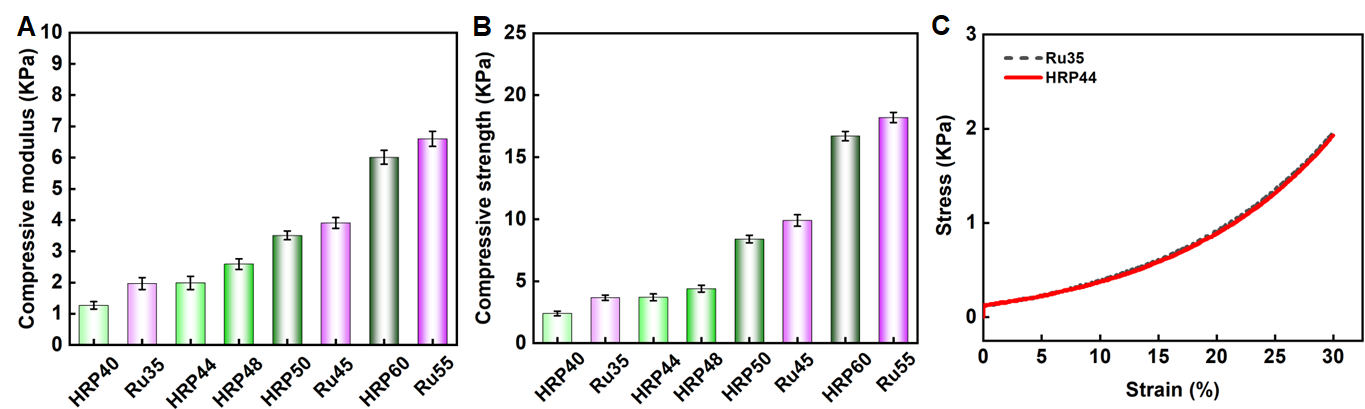
**

**Figure S1** Compressive mechanical features of the SF hydrogels fabricated with varied concentrations of HRP or Ru. (A) Compressive modulus; (B) Compressive strength; (C) Stress-strain curves of the SF hydrogels fabricated with HRP44 and Ru35.

**Table S2** Precursor solution formulations of the hydrogels (H-I ~ H-V)

| Sample code | *V*(SF)/mL | *V*(Ru)/ μL | *V*(SPS)/μL | *V*(HRP)/μL | *V*(H_2_O_2_)/μL |
| --- | --- | --- | --- | --- | --- |
| H-Ⅰ | 2 | 35 | 35 | / | / |
|  |  | (0.85×10^-3^ mol/L) | (0.85×10^-2^ mol/L) |  |  |
| H-Ⅱ | 2 | 26.35 | 26.25 | 11 | 5.5 |
|  |  | (0.63×10^-3^ mol/L) | (0.63×10^-2^ mol/L) | (5.32 U/mL) | (0.13×10^-2^ mol/L) |
| H-Ⅲ | 2 | 17.5 | 17.5 | 22 | 11 |
|  |  | (0.42×10^-3^ mol/L) | (0.42×10^-2^ mol/L) | (10.64 U/mL) | (0.26×10^-2^ mol/L) |
| H-Ⅳ | 2 | 8.75 | 8.75 | 33 | 16.5 |
|  |  | (0.21×10^-3^ mol/L) | (0.21×10^-2^ mol/L) | (15.97 U/mL) | (0.39×10^-2^ mol/L) |
| H-Ⅴ | 2 | / | / | 44 | 22 |
|  |  |  |  | (21.30 U/mL) | (0.52×10^-2^ mol/L) |

Notes：The concentration of SF solution is 3 wt%; the initial concentration of HRP solution is 1000 U/mL；the initial concentration of H_2_O_2_ is 0.49M；the initial concentration of Ru is 0.05M；the initial concentration of SPS is 0.5M.

**Table S3** RT-PCR primer sequences of the chondrogenic characteristic genes

| Gene Name | Forward Primer (5'-3') | Reverse Primer (5'-3') |
| --- | --- | --- |
| ACAN | ATCTATCGCTGTGAAGTGATG | CTCGGTCAAAGTCCAGTGT |
| Col Ⅱ | GGCGAGTCTTGCGTCTAC | GTGCTTCTTCTCCTTGCTCTT |
| PRG4 | GTATTCCCTCTCCCATTGAC | GATACCCAGCATCCATTACAT |
| COMP | ACTGGGTGGTGCTCAATCA | ATCGGTGGCGGTGTTTAC |
| GAPDH | TGTTCCTACCCCCAATGTAT | TTCACCACCTCCTTGATGTC |


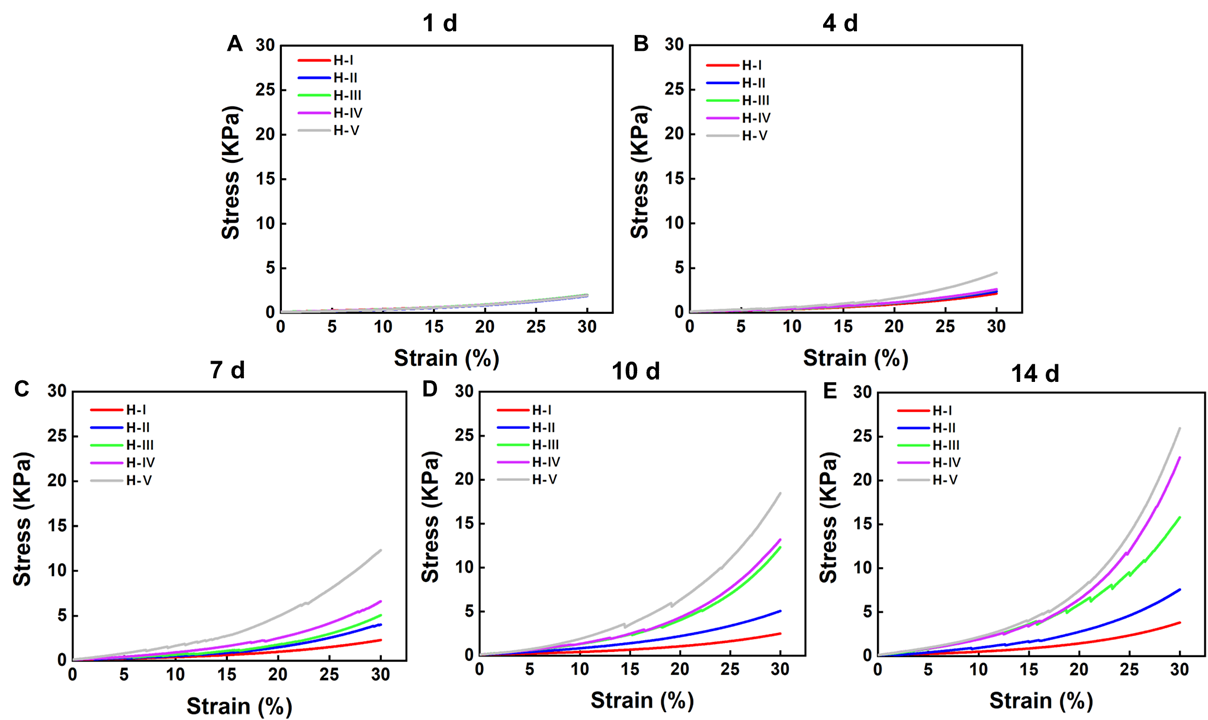


**Figure S2** Stress-strain curves of the fabricated hydrogels during the corresponding conformational transition processes.

**Table S4** The *p* value of cell viability between different SF hydrogels after 1 d of culture in Fig. 6A

|  | H-Ⅰ | H-Ⅱ | H-Ⅲ | H-Ⅳ | H-Ⅴ |
| --- | --- | --- | --- | --- | --- |
| H-Ⅰ | / | Δ  8.21×10^-1^ | Δ  8.76×10^-1^ | Δ  8.67×10^-1^ | Δ  7.51×10^-1^ |
| H-Ⅱ | Δ  8.21×10^-1^ | / | Δ  7.03×10^-1^ | Δ  9.53×10^-1^ | Δ  5.89×10^-1^ |
| H-Ⅲ | Δ  8.76×10^-1^ | Δ  7.03×10^-1^ | / | Δ  7.47×10^-1^ | Δ  8.76×10^-1^ |
| H-Ⅳ | Δ  8.67×10^-1^ | Δ  9.53×10^-1^ | Δ  7.47×10^-1^ | / | Δ  8.21×10^-1^ |
| H-Ⅴ | Δ  7.51×10^-1^ | Δ  5.89×10^-1^ | Δ  8.76×10^-1^ | Δ  8.21×10^-1^ | / |

“**Δ**”: *p* > 0.05, no significant difference

**Table S5** The *p* value of cell viability between different SF hydrogels after 4 d of culture in Fig. 6A

|  | H-Ⅰ | H-Ⅱ | H-Ⅲ | H-Ⅳ | H-Ⅴ |
| --- | --- | --- | --- | --- | --- |
| H-Ⅰ | / | *  3.68×10^-2^ | ***  1.63×10^-7^ | ***  8×10^-9^ | ***  1.15×10^-6^ |
| H-Ⅱ | *  3.68×10^-2^ | / | ***  5.59×10^-6^ | ***  1.54×10^-7^ | ***  5.86×10^-5^ |
| H-Ⅲ | ***  1.63×10^-7^ | ***  5.59×10^-6^ | / | *  3.4×10^-2^ | ***  1.63×10^-7^ |
| H-Ⅳ | ***  8×10^-9^ | ***  1.54×10^-7^ | *  3.4×10^-2^ | / | **  2.37×10^-3^ |
| H-Ⅴ | ***  1.15×10^-6^ | ***  5.86×10^-5^ | ***  1.63×10^-7^ | **  2.37×10^-3^ | / |

“*”: 0.01 < *p* < 0.05, significant difference; “**”: 0.001 < *p* < 0.01, significant difference; “***”: *p* < 0.001, significant difference

**Table S6** The *p* value of cell viability between different SF hydrogels after 7 d of culture in Fig. 6A

|  | H-Ⅰ | H-Ⅱ | H-Ⅲ | H-Ⅳ | H-Ⅴ |
| --- | --- | --- | --- | --- | --- |
| H-Ⅰ | / | ***  9.54×10^-9^ | ***  5.21×10^-11^ | ***  1.32×10^-11^ | ***  1.70×10^-12^ |
| H-Ⅱ | ***  9.54×10^-9^ | / | ***  1.22×10^-4^ | ***  6.10×10^-6^ | ***  9.11×10^-8^ |
| H-Ⅲ | ***  5.21×10^-11^ | ***  1.22×10^-4^ | / | *  1.1×10^-2^ | ***  4.99×10^-4^ |
| H-Ⅳ | ***  1.32×10^-11^ | ***  6.10×10^-6^ | *  1.1×10^-2^ | / | *  1.45×10^-2^ |
| H-Ⅴ | ***  1.70×10^-12^ | ***  9.11×10^-8^ | ***  4.99×10^-4^ | *  1.45×10^-2^ | / |

“*”: 0.01 < *p* < 0.05, significant difference; “***”: *p* < 0.001, significant difference

**Table S7** The *p* value of ACAN expressions between different SF hydrogels after 6 d of culture in Fig. 7C

|  | H-Ⅰ | H-Ⅱ | H-Ⅲ | H-Ⅳ | H-Ⅴ |
| --- | --- | --- | --- | --- | --- |
| H-Ⅰ | / | *  3.10×10^-2^ | ***  1.58×10^-5^ | **  3.61×10^-3^ | *  1.71×10^-2^ |
| H-Ⅱ | *  3.10×10^-2^ | / | ***  1.27×10^-6^ | ***  9.05×10^-5^ | Δ  7.41×10^-1^ |
| H-Ⅲ | ***  1.58×10^-5^ | ***  1.27×10^-6^ | / | **  2.72×10^-3^ | ***  5.21×10^-11^ |
| H-Ⅳ | **  3.61×10^-3^ | ***  9.05×10^-5^ | **  2.72×10^-3^ | / | ***  9.51×10^-7^ |
| H-Ⅴ | *  1.71×10^-2^ | Δ  7.41×10^-1^ | ***  5.21×10^-11^ | ***  9.51×10^-7^ | / |

“**Δ**”: *p* > 0.05, no significant difference; “*”: 0.01 < *p* < 0.05, significant difference; “**”: 0.001 < *p* < 0.01, significant difference; “***”: *p* < 0.001, significant difference

**Table S8** The *p* value of Col Ⅱ expressions between different SF hydrogels after 6 d of culture in Fig. 7D

|  | H-Ⅰ | H-Ⅱ | H-Ⅲ | H-Ⅳ | H-Ⅴ |
| --- | --- | --- | --- | --- | --- |
| H-Ⅰ | / | ***  9.52×10^-4^ | ***  1.00×10^-5^ | **  1.56×10^-3^ | **  1.16×10^-3^ |
| H-Ⅱ | **  9.52×10^-4^ | / | ***  6.29×10^-7^ | Δ  3.44×10^-1^ | Δ  5.62×10^-1^ |
| H-Ⅲ | ***  1.00×10^-5^ | ***  6.29×10^-7^ | / | ***  2.81×10^-7^ | ***  4.54×10^-7^ |
| H-Ⅳ | **  1.56×10^-3^ | Δ  3.44×10^-1^ | ***  2.81×10^-7^ | / | Δ  7.02×10^-2^ |
| H-Ⅴ | **  1.16×10^-3^ | Δ  5.62×10^-1^ | ***  4.54×10^-7^ | Δ  7.02×10^-2^ | / |

“**Δ**”: *p* > 0.05, no significant difference; “**”: 0.001 < *p* < 0.01, significant difference; “***”: *p* < 0.001, significant difference

**Table S9** The *p* value of PRG4 expressions between different SF hydrogels after 6 d of culture in Fig. 7E

|  | H-Ⅰ | H-Ⅱ | H-Ⅲ | H-Ⅳ | H-Ⅴ |
| --- | --- | --- | --- | --- | --- |
| H-Ⅰ | / | Δ  9.69×10^-1^ | *  3.99×10^-2^ | Δ  4.90×10^-1^ | ***  7.82×10^-4^ |
| H-Ⅱ | Δ  9.69×10^-1^ | / | *  4.23×10^-2^ | Δ  4.67×10^-1^ | ***  7.38×10^-4^ |
| H-Ⅲ | *  3.99×10^-2^ | *  4.23×10^-2^ | / | *  3.31×10^-2^ | ***  6.89×10^-5^ |
| H-Ⅳ | Δ  4.90×10^-1^ | Δ  4.67×10^-1^ | *  3.31×10^-2^ | / | ***  2.39×10^-4^ |
| H-Ⅴ | ***  7.82×10^-4^ | ***  7.38×10^-4^ | ***  6.89×10^-5^ | ***  2.39×10^-4^ | / |

“**Δ**”: *p* > 0.05, no significant difference; “*”: 0.01 < *p* < 0.05, significant difference; “***”: *p* < 0.001, significant difference

**Table S10** The *p* value of COMP expressions between different SF hydrogels after 6 d of culture in Fig. 7F

|  | H-Ⅰ | H-Ⅱ | H-Ⅲ | H-Ⅳ | H-Ⅴ |
| --- | --- | --- | --- | --- | --- |
| H-Ⅰ | / | Δ  5.27×10^-1^ | Δ  4.68×10^-1^ | ***  7.22×10^-5^ | ***  1.54×10^-5^ |
| H-Ⅱ | Δ  5.27×10^-1^ | / | Δ  1.89×10^-1^ | ***  3.22×10^-5^ | ***  7.56×10^-6^ |
| H-Ⅲ | Δ  4.68×10^-1^ | Δ  1.89×10^-1^ | / | ***  1.95×10^-4^ | ***  3.69×10^-5^ |
| H-Ⅳ | ***  7.22×10^-5^ | ***  3.22×10^-5^ | ***  1.95×10^-4^ | / | Δ  2.25×10^-1^ |
| H-Ⅴ | ***  1.54×10^-5^ | ***  7.56×10^-6^ | ***  3.69×10^-5^ | Δ  2.25×10^-1^ | / |

“**Δ**”: *p* > 0.05, no significant difference “***”: *p* < 0.001, significant difference

**Table S11** The *p* value of ACAN expressions between different SF hydrogels after 12 d of culture in Fig. 8C

|  | H-Ⅰ | H-Ⅱ | H-Ⅲ | H-Ⅳ | H-Ⅴ |
| --- | --- | --- | --- | --- | --- |
| H-Ⅰ | / | **  5.27×10^-3^ | *  4.49×10^-2^ | **  5.66×10^-3^ | ***  1.54×10^-4^ |
| H-Ⅱ | **  5.27×10^-3^ | / | *  2.81×10^-2^ | ***  9.45×10^-4^ | ***  1.08×10^-5^ |
| H-Ⅲ | Δ  4.49×10^-2^ | *  2.81×10^-2^ | / | ***  2.68×10^-4^ | ***  2.53×10^-4^ |
| H-Ⅳ | *  5.66×10^-3^ | **  9.45×10^-4^ | **  2.68×10^-4^ | / | **  1.46×10^-3^ |
| H-Ⅴ | ***  1.54×10^-4^ | ***  1.08×10^-5^ | ***  2.53×10^-4^ | **  1.46×10^-3^ | / |

“**Δ**”: *p* > 0.05, no significant difference; “*”: 0.01 < *p* < 0.05, significant difference; “**”: 0.001 < *p* < 0.01, significant difference; “***”: *p* < 0.001, significant difference

**Table S12** The *p* value of Col Ⅱ expressions between different SF hydrogels after 12 d of culture in Fig. 8D

|  | H-Ⅰ | H-Ⅱ | H-Ⅲ | H-Ⅳ | H-Ⅴ |
| --- | --- | --- | --- | --- | --- |
| H-Ⅰ | / | ***  4.80×10^-8^ | ***  1.13×10^-5^ | **  4.23×10^-3^ | Δ  2.88×10^-1^ |
| H-Ⅱ | ***  4.80×10^-8^ | / | ***  7.23×10^-5^ | ***  2.37×10^-8^ | ***  2.37×10^-8^ |
| H-Ⅲ | ***  1.13×10^-5^ | ***  7.23×10^-5^ | / | **  1.4410^-3^ | ***  3.54×10^-6^ |
| H-Ⅳ | **  4.23×10^-3^ | ***  2.37×10^-8^ | **  1.4410^-3^ | / | ***  7.19×10^-4^ |
| H-Ⅴ | Δ  2.88×10^-1^ | ***  2.37×10^-8^ | ***  3.54×10^-6^ | ***  7.19×10^-4^ | / |

“**Δ**”: *p* > 0.05, no significant difference; “**”: 0.001 < *p* < 0.01, significant difference; “***”: *p* < 0.001, significant difference

**Table S13** The *p* value of COMP expressions between different SF hydrogels after 12 d of culture in Fig. 8E

|  | H-Ⅰ | H-Ⅱ | H-Ⅲ | H-Ⅳ | H-Ⅴ |
| --- | --- | --- | --- | --- | --- |
| H-Ⅰ | / | ***  3.60×10^-5^ | Δ  6.90×10^-1^ | *  4.90×10^-2^ | ***  2.03×10^-4^ |
| H-Ⅱ | ***  3.60×10^-5^ | / | ***  2.24×10^-5^ | ***  5.50×10^-4^ | ***  1.71×10^-7^ |
| H-Ⅲ | Δ  6.90×10^-1^ | ***  2.24×10^-5^ | / | *  3.47×10^-2^ | ***  3.61×10^-4^ |
| H-Ⅳ | *  4.90×10^-2^ | ***  5.50×10^-4^ | *  3.47×10^-2^ | / | ***  1.61×10^-5^ |
| H-Ⅴ | ***  2.03×10^-4^ | ***  1.71×10^-7^ | ***  3.61×10^-4^ | ***  1.61×10^-5^ | / |

“**Δ**”: *p* > 0.05, no significant difference; “*”: 0.01 < *p* < 0.05, significant difference; “***”: *p* < 0.001, significant difference

**Table S14** The *p* value of PRG4 expressions between different SF hydrogels after 12 d of culture in Fig. 8F

|  | H-Ⅰ | H-Ⅱ | H-Ⅲ | H-Ⅳ | H-Ⅴ |
| --- | --- | --- | --- | --- | --- |
| H-Ⅰ | / | ***  4.38×10^-6^ | **  2.94×10^-3^ | *  1.82×10^-2^ | ***  6.27×10^-7^ |
| H-Ⅱ | ***  4.38×10^-6^ | / | ***  1.53×10^-7^ | ***  3.52×10^-7^ | Δ  6.03×10^-2^ |
| H-Ⅲ | **  2.94×10^-3^ | ***  1.53×10^-7^ | / | *  4.21×10^-2^ | ***  3.58×10^-8^ |
| H-Ⅳ | *  1.82×10^-2^ | ***  5.50×10^-4^ | *  4.21×10^-2^ | / | ***  7.36×10^-8^ |
| H-Ⅴ | ***  6.27×10^-7^ | Δ  6.03×10^-2^ | ***  3.58×10^-8^ | ***  7.36×10^-8^ | / |

“**Δ**”: *p* > 0.05, no significant difference; “*”: 0.01 < *p* < 0.05, significant difference; “**”: 0.001 < *p* < 0.01, significant difference; “***”: *p* < 0.001, significant difference
