# Supplementary material for: Protein conformational transition microenvironment in silk fibroin hydrogels: proliferation and chondrogenesis of encapsulated stem cells
Source: Regen Biomater. 2025 Oct 1;12:rbaf102. doi: 10.1093/rb/rbaf102 (PMC12598285; doi:10.1093/rb/rbaf102)
Supplement: rbaf102_Supplementary_Data [file rbaf102_supplementary_data.zip › Response to Reviewers-20250817 final.docx]

**Responses to the comments of Reviewers**

**Journal n****ame and manuscript number:** Regenerative Biomaterials, RB-2025-314

**Title:** Protein conformational transition microenvironment in silk fibroin hydrogels: Proliferation and chondrogenesis of encapsulated stem cells

**Authors:** Weikun Zhao, Guolong Cai, Jiayao Qian, Jingjing Geng, Xiang Yao*, Yaopeng Zhang*

Reviewers’ comments are in BLUE.

Answers/replies are in BLACK

Text changes in the revised manuscript are in RED.

**Replies to Reviewer #1**

**Comment 1:** This manuscript developed a strategy based on altering the uniformity degrees of crosslinking points for constructing SF hydrogels with equivalent initial properties but different conformational transition rates. Based on this powerful material platform, it first precisely revealed the conformational transition microenvironment on encapsulated cell behaviors under eliminating the possible interference of the varied initial hydrogel features as much as possible. Encapsulated stem cells responses accurately revealed that a relative slow conformational transition rate was more beneficial for cell proliferation, whereas a moderate transition rate was more favorable for chondrogenic differentiation. Related research provided valuable and novel insights for designing effective SF and other protein-based hydrogel biomaterials, which could be well match the journal scope and standard of Regenerative Biomaterials. The following suggestions need to be considered:

For the cell proliferation within 4 days (Figure 6), why the proliferation rate of cells within H-V is faster than that of the cells encapsulated in H-III and H-IV with slower conformational transition? More comprehensive and in-depth discussions should be provided.

**Response:** Thanks for the positive comments and valuable suggestions on our manuscript. As for “the proliferation rate of cells within H-V is faster than that of the cells encapsulated in H-III and H-IV with slower conformational transition within 4 days”, it’s mainly attributed to that the pore size distribution of H-V is wider than H-III and H-IV, thus some “large sized” pores exist in this hydrogel, as shown in **Figure 4**. Although there is a more obvious pore size decreasing trend in H-V, the pore structure still remains intact within the initial 4 days (see **Figure 4**), and these decreased “large sized” pores could still well support cell proliferation than H-III and H-IV. Moreover, the more obvious and suitable material stiffening process of H-V during the initial 4 days (see **Figure 5**) might also favorable for cell proliferation than H-III and H-IV.

Reminded by the kind suggestion. We have provided more comprehensive and in-depth discussions about this phenomenon in the 4^th^ paragraph of Section 3.5. For the convenience of the reviewer and the editor, we copy the modified contents as follows:

“…It showed that the MSCs in H-V exhibited higher cell proliferation rate than those in H-III and H-IV. That’s probably because that the pore size distribution of H-V is wider than H-III and H-IV, thus some “large sized” pores exist in this hydrogel (see **Figure 4**). Although there is a more obvious pore size decreasing trend in H-V, the pore structure still remains intact within the initial 4 days, as shown in **Figure 4**, and these decreased “large sized” pores could still well support cell proliferation than H-III and H-IV. In addition, the more obvious and suitable material stiffening process of H-V during the initial 4 days (see **Figure 5**) might also favorable for cell proliferation than H-III and H-IV….”

**Comment 2:** In Section 3.6, related references to support the descriptions of “The relative intensity of band coloration in the immunoassay exhibited a positive correlation with the expression levels of the indicated proteins, wherein darker bands reflect to higher protein expressions” should be cited.

**Response:** Thanks for the kind suggestions on our manuscript. We have cited 2 related references (Ref. 52 and Ref. 53) to support the mentioned descriptions in Section 3.6.

**Comment 3:** The description of “…which enabled by modulating the uniformity of the chemical crosslinking points while fixing the same crosslinking density” in the Abstract should be changed as “…which enabled by modulating the uniformity of the chemical crosslinking points while fixing the similar crosslinking density”.

**Response:** Thanks for this kind comment. We have changed the corresponding contents as “… which enabled by modulating the uniformity of the chemical crosslinking points while fixing the similar crosslinking density.” in the Abstract.

**Comment 4:** In the last paragraph of Introduction, the statement of “Ru disperses more easily and uniformly into the hydrogel precursor solution compared to HRP” should be changed as “Ru disperses more easily and uniformly into the hydrogel precursor system compared to HRP”.

**Response:** Thanks for the valuable suggestion. Remined by this comment and other reviewers’ suggestions, we have changed the corresponding contents as “Ru could disperse more easily and uniformly into the hydrogel precursor system and triggering the reaction much quickly compared to HRP” in the last paragraph of Introduction.

**Comment 5:** Please carefully check the text format of “H-I ~ H-V” through the whole manuscript.

**Response:** Thanks for this kind suggestion. We have carefully checked the text format of “H-I ~ H-V” through the whole manuscript and changed them accordingly.

**Comment 6:** The recent studies of hydrogels for cell delivery and bioprinting in chondrogenesis are suggested to be referred, such as:

Interdisciplinary Materials, 2024;3:738-756. DOI: 10.1002/idm2.12181

Smart Materials in Medicine, 2022;3: 85-93. DOI: 10.1016/j.smaim.2021.12.002

**Response:** Thanks for the kind comment. We have carefully studied the mentioned literatures and cited them as Ref. 29 and Ref. 21 in the 1^st^ paragraph of Introduction.

**Replies to Reviewer #2**

**Comment 1:** In this report, Zhao et al. have developed a novel SF hydrogel platform for systematically investigating the effects of protein conformational transition microenvironment on the hydrogel encapsulated stem cell behaviors. The unique feature of this material platform lies in that the fabricated hydrogels showed equivalent initial properties but different conformational transition rates. Further results showed that this unique dynamical material cue could have significant effects on cell proliferation and chondrogenesis. This report has provided some valuable references and new hints for the construction of effective cartilage tissue engineering scaffolds or cartilage organoids. To further improve the manuscript quality, some issues need to be addressed.

Compared with the reported influence of the conformational transition of SF based hydrogels coupled with different initial material features on the chondrogenic differentiation of encapsulated stem cells, what deeper or confirmed insights has this study further afforded? The authors are advised to expand some relevant discussions.

**Response:** Thanks for the positive comments and valuable suggestions on our manuscript. Reminded by this comment, we have expanded the relevant discussions in the penultimate paragraph of Section 3.6. For the convenience of the reviewer and the editor, we copy the expanded contents as follows:

“In the related field, the reported influence of the conformational transition of SF based hydrogels coupled with different initial hydrogel features on cell differentiation have also hinted that moderate conformational transition could promote the chondrogenesis of stem cells[14, 23]. Compared with them, the current report has accurately confirmed that the moderate microenvironment of material stiffening/shrinkage induced by appropriate conformational transition exerts significant enhancing effects on stem cell chondrogenesis, as it minimizes the possible interference of the varied initial hydrogel features as much as possible. Together, as for cell chondrogenesis, this report could further hint that these novel dynamical material cues induced by conformation transition may be much stronger than other static material cues, such as the chemical composition[23] and initial modulus[14]. This may also explain why optimal chondrogenesis consistently occurred on SF-based hydrogels with moderate conformational transition rates, even when using different material platform construction strategies[14, 23].”

**Comment 2:** In the Introduction, the description of “…have demonstrated that such material properties, particularly the pore size and modulus, significantly influence cell behaviors such as adhesion, proliferation…” should be changed as “…have demonstrated that such material properties, particularly the pore size and modulus, significantly influenced cell behaviors such as adhesion, proliferation…”

**Response:** Thanks for the kind comment. We have changed the corresponding contents as “…have demonstrated that such material properties, particularly the pore size and modulus, significantly influenced cell behaviors such as adhesion, proliferation…” in the 1^st^ paragraph of Introduction.

**Comment 3:** Cell proliferation responses between 4 days and 7 days of cell culture have showed a little different trend, especially the group of H-V between them. The authors are advised to provide more deep discussions.

**Response:** Thanks for the kind comments. The different cell proliferation trend between 4 days and 7 days of cell culture lies in that “the proliferation rate of cells within H-V is faster than that of the cells encapsulated in H-III and H-IV with slower conformational transition within 4 days” (see **Figure 6A**). As for this difference, it’s mainly attributed to that the pore size distribution of H-V is wider than H-III and H-IV, thus some “large sized” pores exist in this hydrogel, as shown in **Figure 4**. Although there is a more obvious pore size decreasing trend in H-V, the pore structure still remains intact within the initial 4 days (see **Figure 4**), and these decreased “large sized” pores could still well support cell proliferation than H-III and H-IV. Moreover, the more obvious and suitable material stiffening process of H-V during the initial 4 days (see **Figure 5**) might also favorable for cell proliferation than H-III and H-IV.

Reminded by the kind comments. We have afforded more in-depth discussions about this issue in the 4^th^ paragraph of Section 3.5. For the convenience of the reviewer and the editor, we copy the modified contents as follows:

“…It showed that the MSCs in H-V exhibited higher cell proliferation rate than those in H-III and H-IV. That’s probably because that the pore size distribution of H-V is wider than H-III and H-IV, thus some “large sized” pores exist in this hydrogel (see **Figure 4**). Although there is a more obvious pore size decreasing trend in H-V, the pore structure still remains intact within the initial 4 days, as shown in **Figure 4**, and these decreased “large sized” pores could still well support cell proliferation than H-III and H-IV. In addition, the more obvious and suitable material stiffening process of H-V during the initial 4 days (see **Figure 5**) might also favorable for cell proliferation than H-III and H-IV….”

**Comment 4:** The statement of “The corresponding SF hydrogel systems reported thus far fail to meet the requirement for equivalent initial properties” should be better changed to “The corresponding SF hydrogel platforms reported thus far fail to meet the requirement for equivalent initial material properties”.

**Response:** Thanks for the kind suggestion. Remined by this comment and other reviewers’ suggestions, we have changed the corresponding contents as “The corresponding SF hydrogel platforms reported thus far fail to meet the requirement for highly similar initial material properties.” in the 4^th^ paragraph of Introduction.

**Replies to Reviewer #3**

**Comment 1:** The work entitled “Protein conformational transition microenvironment in silk fibroin hydrogels: Proliferation and chondrogenesis of encapsulated stem cells” by Weikun Zhao, Prof. Xiang Yao, Prof. Yaopeng Zhang, et al, reported the interesting effects of conformational transition or dynamic conformation changes on the stem cell behaviors. They applied two chemical crosslinking methods to control the crosslinking state (uniformity) and the conformation transition rates. This work follows their previous work (ref [20] Regenerative Biomaterials, 2025, 12, rbaf019.) on controlling the crosslinking density and conformation transition rates. The methodology in the two works is very similar. I believe a more in-depth discussion to address the relationship and the differences between these two works would be needed.

**Response:** Thanks for the kind comment and useful suggestion on our manuscript. Reminded by this comment, we have provided more in-depth discussions between these two different material design and construction methodologies in the 3^rd^~5^th^ paragraphs of Introduction. For the convenience of the reviewer and the editor, we copy the modified contents as follows:

“Until now, a few researchers have attempted to develop SF based hydrogels with varied transition rates of protein conformation. For example, Mahajan *et al.* [23] created several composite hydrogels with varied protein conformational transition rates by combining SF with carboxymethyl cellulose and gelatin. However, this approach relies on altering the ratios of SF and other components, leading to significant variations in the initial chemical composition and compressive modulus of the hydrogels. Furthermore, Cai *et al.* [14] developed a strategy based on changing the chemical crosslinking density of hydrogels, using SF as the sole component to construct hydrogels with varied transition rates of protein conformation. This reported strategy[14] effectively eliminates the interference of different initial chemical components, while the different crosslinking density leads to significant differences in the initial modulus and average pore size of hydrogels. In order to more clearly reveal the conformational transition microenvironments effects on cell behaviors within SF hydrogels, it is crucial to construct SF hydrogels with equivalent initial features while different protein conformational transition rates.”

“The corresponding SF hydrogel platforms reported thus far fail to meet the requirement for highly similar initial material properties. This failure could probably introduce non-negligible interference factors when investigating the impact of conformational transitions on cell behaviors. In the current literature, a novel strategy based on altering the uniformity degrees of crosslinking points while fixing the crosslinking density was proposed for constructing the desired SF hydrogel platforms with highly similar initial material features but different protein conformational transition rates, as schematically illustrated in **Figure 1A**….”

“…The key difference between these two systems is that the molecular weight of HRP (Mw = 44,000 Da) is quite larger than that of Ru (Mw = 750 Da)[33]. And the reaction speed of Ru/SPS system is significantly faster than that of the HRP/H_2_O_2_ system[3]. Comprehensively, under the similar hydrogel fabrication condition, Ru could disperse more easily and uniformly into the hydrogel precursor system and triggering the reaction much quickly compared to HRP, leading to a crosslinking network with a higher degree of uniformity. Furthermore, through the combination and dosage ratio regulation of Ru/SPS and HRP/H_2_O_2_, SF hydrogels with similar crosslinking density while different crosslinking uniformity degrees are expected to be constructed. Be different form our previous literature[14] and other related report[23], this hydrogel construction strategy aims to minimize the impacts of initial material properties differences as much as possible, thus provides a robust material platform for accurately studying and confirming the effects of conformational transition microenvironments on encapsulated stem cell behaviors. The related researches are anticipated to serve valuable reference for the controlling of conformational transition within SF hydrogels, and also offer important insights for the effective application of protein conformational transition in 3D cell culture, organoid construction and tissue repair.”

In addition, we have also provided more in-depth discussions about the differences and similarities in cell proliferation and differentiation responses between these two literatures in the last paragraph of Section 3.5 and the penultimate paragraph of Section 3.6. For the convenience of the reviewer and the editor, we copy the expanded contents as follows:

“When eliminating the possible interference of the varied initial hydrogel features as much as possible, the current report has proved that the SF hydrogel with slower conformational transition rate was more favorable for stem cell proliferation. Accompanied with different modulus and pore size features, our previous report[14] has illustrated that the hydrogel with moderate conformational transition rate was more suitable for cell proliferation. The different cell response trends could probably own to that the important cues for effecting cell proliferation in the previous report[14] not only contained the varied dynamical protein conformational transition, but also the different initial hydrogel modulus and pore sizes induced by different hydrogel crosslinking densities. Summarily, the conformational transition microenvironment within SF hydrogels could effectively regulate encapsulated cell proliferation. All the hydrogels could well support cell proliferation and the group with slower conformational transition rate was more favorable for cell proliferation when eliminating the possible interference of the varied initial hydrogel characteristics as much as possible. The phenomenon was probably own to the integrated effects of pore structure shrinkage and material stiffening process caused by the varied conformational transition microenvironments.”

“…In the related field, the reported influence of the conformational transition of SF based hydrogels coupled with different initial hydrogel features on cell differentiation have also hinted that moderate conformational transition could promote the chondrogenesis of stem cells[14, 23]. Compared with them, the current report has accurately confirmed that the moderate microenvironment of material stiffening/shrinkage induced by appropriate conformational transition exerts significant enhancing effects on stem cell chondrogenesis, as it minimizes the possible interference of the varied initial hydrogel features as much as possible. Together, as for cell chondrogenesis, this report could further hint that these novel dynamical material cues induced by conformation transition may be much stronger than other static material cues, such as the chemical composition[23] and initial modulus[14]. This may also explain why optimal chondrogenesis consistently occurred on SF-based hydrogels with moderate conformational transition rates, even when using different material platform construction strategies[14, 23]. ”

**Comment 2:** Other questions for the authors to consider are as follows.

The first sentence of the introduction cited 12 references, which is inappropriate. Please revise the sentence to cite more focused literature.

**Response:** Thanks for the kind suggestion. We have modified the mentioned sentence and only retained the most relevant references to support the corresponding contents. The modified contents are as follows:

“Silk fibroin (SF) is a natural protein that can be feasibly produced on a large scale. This raw material has garnered significant interest from biomaterial scientists and entrepreneurs because of its comprehensive excellent performance, including good biocompatibility, adjustable biodegradability, ease of processing, and FDA approved[1-6].”

**Comment 3:** In the methods section, ‘The SF hydrogel (H-I ~ H-V) samples were gelled in a 12-well plate, with each well containing 2 mL of SF hydrogel precursor solution’, appeared multiple times. Would this information be moved to the earlier section of hydrogel preparation?

**Response:** Thanks for the kind suggestion. Reminded by this comment, we have mentioned this method specifically only in Section 2.4.1 when it firstly appeared, and described it as “The SF hydrogels (H-I ~ H-V) were fabricated identically to those in section 2.4.1” when the same method was used at the following sections.

**Comment 4:** The ‘equivalent’ initial state was actually not the same, due to the variance in the porous morphology and mechanical properties. Presumably, the larger variability in the microstructure should also result in larger mechanical properties of the hydrogels. Please give the variability of mechanical properties, e.g. compressive modulus. The word ‘equivalent’ may need to be revised to more precise description of the H-I to H-V hydrogels.

**Response:** Thanks for the kind comment. As for the compressive modulus, the average value of H-I ~ H-V were 3.70±0.05, 3.68±0.10, 3.76±0.12, 3.78±0.10, and 3.70±0.15 kPa, respectively (see **Figure 2D**). The variance of this mechanical property is very small, probably because that the crosslinking densities of these hydrogels are highly similar to each other (see **Figure 2B**). Moreover, statistical results revealed no significant differences in compressive moduli among the five SF hydrogels (*p* > 0.05), confirming that these hydrogels exhibit highly similar initial compressive mechanical properties.

As for the pore structures, the results indicated that H-I has a relatively uniform pore size feature, while the pore size heterogeneity of the hydrogels progressively increased from H-I to H-V. Further quantitative analysis showed that the average pore sizes were 92.4 ± 22.1, 91.5 ± 40.3, 90.1 ± 55.8, 89.7 ± 70.6 and 93.7 ± 88.0 μm, respectively from H-I to H-V (see the column chart in the bottom right corner of **Figure 2E**). Although the variance of the pore size gradually increased, the statistical *p*-values between each group were still all greater than 0.05, suggesting similar initial average pore size exist in these SF hydrogels.

Statistically, the crosslinking density and mechanical property of H-I ~ H-V are highly similar to each other. So, it reasonably speculated that the larger variance of the pore size is probably own to the different uniformity of crosslinking points of these hydrogels.

Remined by this kind comment, we have modified the related descriptions about the initial properties of the hydrogel to make it more clearly and precisely. In addition, for considering the exist variance of the mentioned material properties, we have changed the word “equivalent” to “highly similar” throughout the entire literature to make the descriptions more precisely and objectively. For the convenience of the reviewer and the editor, we copy the main contents as follows:

“…**Figure 2D** showed the corresponding compressive modulus of each hydrogel, which are calculated from the data of **Figure 2C**. Specifically, the average compressive moduli of H-I ~ H-V were 3.70±0.05, 3.68±0.10, 3.76±0.12, 3.78±0.10, and 3.70±0.15 kPa, respectively. Statistical results revealed no significant differences in compressive moduli among the five SF hydrogels (*p* > 0.05), confirming that these hydrogels exhibit highly similar initial compressive mechanical properties.”

“…The statistical results (the column chart in the bottom right corner of **Figure 2E**) showed that the average pore sizes were 92.4 ± 22.1, 91.5 ± 40.3, 90.1 ± 55.8, 89.7 ± 70.6 and 93.7 ± 88.0 μm, respectively from H-I to H-V. On the one hand, the statistical *p*-values between each group were all greater than 0.05, suggesting highly similar initial average pore size exist in these SF hydrogels. On the other hand, the variance of the pore size data gradually increased from H-I to H-V, which indirectly indicated a progressive increase in the crosslinking point uniformity of these hydrogels.”

**Comment 5:** In figure 1, it may be inappropriate to show schematics that already suggested the results on the cell behaviors. Do the authors have direct structure evidence of the variability differences in the crosslinking structure.

**Response:** Thanks for the kind comment. In this literature, Figure 1 is mainly for displaying the schematic presentation of the controlling of protein conformational transition in SF hydrogels and the corresponding effects on encapsulated cell behaviors. In order to emphasize and intuitively reflect the speculated cell behavior differences induced by different conformational transition microenvironments, a schematic diagram for displaying the speculated differential cell responses was drawn. Similar graphic display strategy (material design + desired material effects) is also a commonly used way for “Figure 1” to illustrate the research topic of a functional material related scientific research paper, such as the *Regen. Biomater.,* 2025, 12, rbae131, doi.org/10.1093/rb/rbae131; *Regen. Biomater.,* 2025, 12, rbae134, doi.org/10.1093/rb/rbae134; *Biomaterials*, 2026, 35, 123539, doi.org/10.1016/j.biomaterials.2025.123539; *Biomaterials*, 2026, 325, 123551, doi.org/10.1016/j.biomaterials.2025.123551; *Adv. Mater.,* 2025, 37, 2501838, doi.org/10.1002/adma.202501838; *Adv. Mater.,* 2025, 37, 2419416, doi.org/10.1002/adma.202419416 and *Bioact. Mater.,* 2025, 54, 34-46, doi.org/10.1016/j.bioactmat.2025.07.043, etc.

As for the evidence of the variability differences in the crosslinking structure (especially for the uniformity of crosslinking points) of H-I to H-V, it is quite difficult to obtain the direct evidence based on our current characterization technologies. In this literature, we have used the fluorescence intensity at the characteristic peak of 410 nm to directly reflect the crosslinking density (di-tyrosine bonds as the chemical crosslinking points) of the fabricated hydrogels, which illustrated that the crosslinking density of the SF hydrogels is highly similar to each other (see the 2^nd^ paragraph of Section 3.1). In addition, the statistical results (the column chart in the bottom right corner of **Figure 2E**) showed that the average pore sizes were 92.4 ± 22.1, 91.5 ± 40.3, 90.1 ± 55.8, 89.7 ± 70.6 and 93.7 ± 88.0 μm, respectively from H-I to H-V. On the one hand, the statistical *p*-values between each group were all greater than 0.05, suggesting highly similar initial average pore size exist in these SF hydrogels. On the other hand, the variance of the pore size data gradually increased from H-I to H-V, which indirectly indicated a progressive increase in the crosslinking point uniformity of these hydrogels (see the 4^th^ paragraph of Section 3.1). Moreover, the special pore size features of H-V with highest variance could well explain the “abnormal” cell proliferation behaviors within this hydrogel. It showed that the MSCs in H-V exhibited higher cell proliferation rate than those in H-III and H-IV during 1~ 4 days of culture. That’s probably because that the pore size distribution of H-V is wider than H-III and H-IV, thus some “large sized” pores exist in this hydrogel (see **Figure 4**). Although there is a more obvious pore size decreasing trend, the pore structure still remains intact in H-V within the initial 4 days, as shown in **Figure 4**, and these decreased “large sized” pores could still well support cell proliferation than H-III and H-IV (see the 4^th^ paragraph of Section 3.5).

**Together, these results and analysis could indirectly support the differential uniformity of crosslinking points of these hydrogels from different perspectives. In order to make the contents more clearly, we have modified the related descriptions in Sections 3.1 and 3.5.** For the convenience of the reviewer and the editor, we copy the modified contents as follows:

“…**Figure 2A** showed the characterized fluorescence spectra of the indicated hydrogels, which could be used to reflect the di-tyrosine bonds contents of the hydrogels (H-I ~ H-V), it indicated that the spectra are relatively similar between each other. Based on these spectra, we have also calculated and presented the statistical results of the fluorescence intensity at the characteristic peak of 410 nm[39], as shown in **Figure 2B.** It demonstrated that no significant difference could be found among these five kinds of hydrogels, which suggest that these fabricated hydrogels (H-I ~ H-V) have highly similar initial crosslinking density.”

“…The statistical results (the column chart in the bottom right corner of **Figure 2E**) showed that the average pore sizes were 92.4 ± 22.1, 91.5 ± 40.3, 90.1 ± 55.8, 89.7 ± 70.6 and 93.7 ± 88.0 μm, respectively from H-I to H-V. On the one hand, the statistical *p*-values between each group were all greater than 0.05, suggesting highly similar initial average pore size exist in these SF hydrogels. On the other hand, the variance of the pore size data gradually increased from H-I to H-V, which indirectly indicated a progressive increase in the crosslinking point uniformity of these hydrogels.”

“…The data in **Figure 6B** (LU_4d_/LU_1d_) for evaluation cell proliferation during 1~ 4 days basically conform to this rule except for the group of H-V. It showed that the MSCs in H-V exhibited higher cell proliferation rate than those in H-III and H-IV. That’s probably because that the pore size distribution of H-V is wider than H-III and H-IV, thus some “large sized” pores exist in this hydrogel (see **Figure 4**). Although there is a more obvious pore size decreasing trend in H-V, the pore structure still remains intact within the initial 4 days, as shown in **Figure 4**, and these decreased “large sized” pores could still well support cell proliferation than H-III and H-IV. In addition, the more obvious and suitable material stiffening process of H-V during the initial 4 days (see **Figure 5**) might also favorable for cell proliferation than H-III and H-IV….”

**Comment 6:** The explanation for the induced variability in the microstructure corresponding the content of HRP+H_2_O_2_, appeared not so convincing. Usually we dissolve HRP in water with required contents, and then mix the HRP solution with RSF solution to reach equilibrium before introducing H_2_O_2_ for reactions. Therefore, it won’t have an issue of MW-related diffusivity problem.

**Response:** In this literature, two crosslinking systems both rely on mild chemical reactions to form di-tyrosine as hydrogel crosslinking points were chosen to fabricated SF hydrogels. One is a visible light inducing crosslinking system using tris(2,2-bipyridyl) dichlororuthenium(II) hexahydrate (Ru) and sodium persulfate (SPS). The other is a body temperature (37℃) inducing crosslinking system using horseradish peroxidase (HRP) and hydrogen peroxide (H_2_O_2_). The specifical procedure in this literature is descripted as that “…Ru, SPS, HRP and H_2_O_2_ solutions were added to the SF solution. After thoroughly mixing, the crosslinking was carried out at 37°C under white light illumination (intensity: 30 mW cm⁻^2^) for 40 min to form the HRP-SF or Ru-SF hydrogels”, see Section 2.3.1. The key difference between these two systems is that the molecular weight of HRP (Mw = 44,000 Da) is quite larger than that of Ru (Mw = 750 Da). Except for the M_W_ difference of HRP and Ru, the other difference is that the reaction speed of Ru/SPS system is significantly faster than that of the HRP/H_2_O_2_ system. So, under the similar hydrogel fabrication condition, Ru could disperse more easily and uniformly into the hydrogel precursor system and triggering the reaction much quickly compared to HRP, leading to a crosslinking network with a higher degree of uniformity (which will inducing slower conformational transition rate).

In addition, another published literature (*Adv Healthc Mater* 2020; 9:1901667., Ref. 33) has also hinted that the conformational transition rate of HRP/H_2_O_2_ induced SF hydrogels was quite faster than that of the Ru/SPS induced ones. It also speculated that the M_W_-related diffusivity difference might be the main reasons for inducing SF hydrogels with different crosslinking point uniformity. It demonstrated that the Ru/SPS induced SF hydrogels might have higher uniformity of crosslinking points, thus further induced slower conformation transition rate.

Remined by this comment and the above-mentioned analysis, we have modified the related speculations and explanations in the last paragraph of Introduction as follows:

“…The key difference between these two systems is that the molecular weight of HRP (Mw = 44,000 Da) is quite larger than that of Ru (Mw = 750 Da)[33]. And the reaction speed of Ru/SPS system is significantly faster than that of the HRP/H_2_O_2_ system[3]. Comprehensively, under the similar hydrogel fabrication condition, Ru could disperse more easily and uniformly into the hydrogel precursor system and triggering the reaction much quickly compared to HRP, leading to a crosslinking network with a higher degree of uniformity….”

**Comment 7:** Can the authors explain why these hydrogels exhibited varied conformation transition rates. Presumably, the authors left the hydrogels to naturally develop beta-sheet conformations. What was the reason behind the varied conformation transition rates.

**Response:** Natural SF molecules possess a unique structure characterized by alternating hydrophilic and hydrophobic chain segments. And the hydrophobic segments typically composed polypeptide sequences as (-GAGAGS-)_n_ or (-GAGAGY-)_n_, which could easily self-assembled with each other to form *β*-sheet crystalline structures. The inherent hydrophobic and hydrophilic chain arrangement enables spontaneous formation of regularly alternating crystalline domains (*β*-sheet structures) and amorphous domains (random coil structures) during the natural spinning process. This distinctive organized hierarchical architecture endows natural silk with excellent mechanical strength and toughness.

In this report, the chemical crosslinked SF hydrogels are formed through crosslinking between tyrosine in their hydrophilic molecular segments. Consequently, SF molecules in these hydrogels still possess “intact” hydrophobic chain segments. Based on the intrinsic characteristics of natural SF molecules, the free movement and self-assembly of hydrophobic chain segments inevitably induce a conformational transition of the SF molecules from random coil structures to *β*-sheet structures with lower-energy state. This constitutes the fundamental mechanism underlying protein conformational transitions within the fabricated SF hydrogels.

As for the regulation of the conformation transition rate, a unique strategy based on altering the uniformity degree of hydrogel crosslinking points has been proposed in this literature for constructing SF hydrogels with highly similar initial properties but varied transition rates of protein conformation, as schematically illustrated in **Figure 1A**. Under highly similar crosslinking density, the hydrogel with low uniformity (LU) of crosslinking points would exist some relative “longer” hydrophobic molecular segments compared to the hydrogel own high uniformity (HU) of crosslinking points. These “longer” hydrophobic segments are more feasibly to undergo free movement and self-assembly, resulting in faster conformational transition rates in the LU group. What’s more, the highly similar crosslinking density could guarantee highly similar initial material properties of SF hydrogel as much as possible.

Reminded by this kind comment, we have modified the related explanations and discussions to make it more clearly in the 3^rd^ and 4^th^ paragraphs of Section 3.2. For the convenience of the reviewer and the editor, we copy the modified contents as follows:

“As we known, natural SF molecules possess a unique structure composed by alternating hydrophilic and hydrophobic chain segments. And the hydrophobic segments could easily self-assembled with each other to form *β*-sheet crystalline structures. The inherent hydrophobic and hydrophilic chain arrangement enables spontaneous formation of regularly alternating *β*-sheet crystalline domains and amorphous domains during the natural spinning process[4, 42]. This distinctive organized hierarchical architecture endows natural silk with excellent mechanical strength and toughness. In this report, the fabricated SF hydrogels are formed through crosslinking between tyrosine in their hydrophilic molecular segments. Consequently, SF molecules in these hydrogels still possess “intact” hydrophobic chain segments. Based on the intrinsic characteristics of natural SF molecules, the free movement and self-assembly of hydrophobic chain segments inevitably induce a conformational transition of the SF molecules from random coil structures to *β*-sheet structures with lower-energy state[14, 23]. This constitutes the fundamental mechanism underlying protein conformational transitions within the fabricated SF hydrogels.”

“The above-mentioned results proved that the SF hydrogels with high uniform crosslinking points exhibited slower conformational transition, while those with low uniform crosslinking points underwent faster conformational transition. Under similar crosslinking density, the hydrogel with low uniformity of crosslinking points would exist some relative “longer” hydrophobic molecular segments compared to the hydrogel own high uniformity of crosslinking points. The “longer” hydrophobic segments in the hydrogel with low uniformity of crosslinking points are more easily to move freely and self-assemble[14, 33], thereby inducing faster protein conformation transitions. In addition, as the increasing of *β*-sheet contents, the transparency ability and volume of the SF hydrogel decreased[14, 22, 23]. That’s probably why the hydrogels with faster conformational transition also present faster transparency decreasing (**Figure 3B-C**) and size contraction trends (**Figure 3B**).”

**Comment 8:** The authors stated the conformational transition could lead to a decrease in the pore size. This observation in Figure 4 might be due to the ‘artefact’ of freeze-drying of the hydrogels. Stiffer hydrogels with more beta-sheet would restrict the growth of ice crystals, therefore, exhibiting smaller pore size, thicker walls, as less connectivity. How can the authors distinguish the morphological effects and mechanical effects of the microenvironment.

**Response:** Thanks for the kind comment. Except for the evidence of SEM images after freeze-drying of the hydrogels, this report has also provided some effective circumstantial evidences for supporting the pore size decreasing trends along with protein conformational transition without “freeze-drying process”. For example, (1) the macroscopic volume of hydrogels will shrink significantly with the occurrence of conformational transition, and the faster the conformational transition, the more obvious the volume shrinkage (see **Figure 3B**); (2) as the occurrence of conformational transition, the *β*-sheet structure contents within SF hydrogels will obviously increase (see **Figure 3A**), which will significantly increase the physical crosslinking density of the hydrogel, thus decreasing the hydrogel pore size. Summarily, although some features of the hydrogel might influence the formation of ice crystals during freeze-drying process, the trend that the pore size of hydrogel decreases significantly with the occurrence of protein conformational transformation is tenable. And the pore size will decrease more seriously within the hydrogels with faster conformational transition rate. So, the compressive mechanical increasing effect and the dynamical pore size decreasing effect both exist during the protein conformational transition. And this combined stiffening/shrinkage process further significantly influenced stem cell behaviors within these hydrogels.

Reminded by this kind comment, we have modified the related descriptions and discussions to make the contents more clearly in the 2^ed^ paragraphs of Section 3.3. For the convenience of the reviewer and the editor, we copy the modified contents as follows:

“…After 4 days of incubation, the pore sizes have slightly decreased, probably because the slight conformational transition and weak contraction during the early incubation stage. With the extension of incubation time (about 1 week), the pore sizes feature of H-III, H-IV and H-V have obviously decreased compared to their initial state, especially the groups of H-IV and H-V own the fastest conformational transition processes. Furthermore, even the groups with the slowest conformational transition processes (H-I and H-II) showed significant pore size decreasing compared to their initial state when after 10 days to 2 weeks of incubation. As for the pore size decreasing along with protein conformational transition, it probably owns to that the increased *β*-sheet structures within SF hydrogels (see **Figure 3A**) increased the physical crosslinking density and decreased the volume of hydrogels (see **Figure 3B**). At this later stage, some obvious pore structure collapse has also happened on the groups with faster conformational transition rates (H-IV and H-V), mainly because the faster material contraction and the lowest uniformity of the crosslinking points and pore structures.”

**Comment 9:** Figure 7 and 8 show the cell chondrogenesis effects after 6 and 12 days respectively. The time points did not quite correspond to the time points for structure and property characterizations. Can these two figures be merged or one be moved to the SI?

**Response:** Thanks for the kind comment and suggestion on our manuscript. In this literature, we have carefully chosen several time points (relatively dense time points) to characterize the hydrogel features during 1~14 days of incubation to understand the influence of different conformational transition rates on the characteristics changes of SF hydrogels. Furthermore, in order to figure out the effects of the varied protein conformational transition microenvironments on the chondrogenesis of stem cells, we have chosen two typical time points (6 and 12 days) to investigate the mentioned effects during relatively “short” and “long” incubation times, respectively.

When compared the cell differentiation results between 6 days and 12 days of culture, it illustrated that the “cumulative” cartilage specifical proteins expression trends were quite similar to each other, as comprehensively and schematically illustrated in **Figures 7B and 8B.** More specifically, MSCs encapsulated in H-III exhibited the highest expression of cartilage specifical proteins, and the protein expression levels gradually decreased from H-III to H-V and H-III to H-I. While the comprehensive “transient” cartilage specifical genes expression (RT-PCR results) shifting of the highest peak value from H-III in the 6 day results (see **Figures 7C-F**) to H-Ⅱ in the 12 day results (see **Figures 8C-F**). So, some gene expression differences have also existed during relatively “short” and “long” incubation times.

**In order to show the relevant impacts (similarities and differences) on cell chondrogenesis during relatively “short” and “long” incubation times more comprehensively and clearly, we have included both Figure 7 and Figure 8 in the main text of this literature, and also showed them individually.**

To make the related contents more clearly, in-depth discussions for the differences between WB and RT-PCR results at day 12 and also the highest peak value shifting of RT-PCT results from day 6 to day 12 have both been carefully modified in the 7^th^ and 8^th^ paragraphs of Section 3.6. For the convenience of the reviewer and the editor, we copy the modified contents as follows:

“…Compared with the chondrogenesis trend obtain by the WB results (**Figures 8B**), the biggest difference lies in the shifting of the highest peak value from H-III in the WB results to H-II in the RT-PCR results. As mentioned before, be different from WB data, the RT-PCR data only reflects the transient expression levels of genes in cells during a relative short period. Therefore, the revealed peak value difference at day 12 might hint that the chondrogenic ability of cells in H-II was higher than that of the H-III at the very late stage of this period. Because the WB results reflect the cumulative expression level of indicated proteins during 1~12 days, it still presented the highest expression on the H-III, just the same as that presented at day 6, which is the intermediate time point.”

“The genes expression illustrated that H-II has the “transit” optimal chondrogenic induction ability at the very late stage of 1 ~ 12 days (shift from H-III at day 6 to H-II at day 12). The possible reasons are as follows: (1) During the mentioned later stage, the relatively slow conformational transition rate of H-II make its pore size decrease to a suitable range which is favorable for cell chondrogenesis; (2) Since the dynamical material stiffening cues of H-II induced by the corresponding conformational transition at the later stage were quite similar to those of H-III during the medium stage, this suitable dynamical stiffening cue could further elevate stem cell chondrogenesis. As for the H-IV and H-V with faster conformational transition rate, the excessively fast dynamical stiffening process induced by faster conformational transition might be unfavorable for cell chondrogenesis, and the induced excessively small pore size could obviously further restrict cell proliferation and functioning, thus comprehensively causing the weakest cell chondrogenesis. As for H-I with the slowest conformational transition rate, the relatively large pore size (**Figure 4**) and weakest stiffening process (**Figure 5**) were neither useful for cell chondrogenesis, thus also inducing weaker cell chondrogenesis.”

The quality of the manuscript has been improved after revision. We sincerely thank the comments and suggestions from the three reviewers.
